# GelMA-based bioactive hydrogel scaffolds with multiple bone defect repair functions: therapeutic strategies and recent advances

**DOI:** 10.1186/s40824-023-00422-6

**Published:** 2023-09-15

**Authors:** Bixia Zhou, Xulei Jiang, Xinxin Zhou, Wuyuan Tan, Hang Luo, Shaorong Lei, Ying Yang

**Affiliations:** 1 Department of Plastic Surgery, Xiangya Hospital, Central South University, 87 Xiangya Road, Changsha, 410008 Hunan PR China; 2 National Clinical Research Center for Geriatric Disorders, Xiangya Hospital, Central South University, Changsha, 410008 PR China; 3 State Key Laboratory of Powder Metallurgy, Central South University, Changsha, 410083 PR China

**Keywords:** Bioactive scaffolds, GelMA, Composite hydrogels, Bone defects, Bone regeneration

## Abstract

Currently, the clinical treatment of critical bone defects attributed to various causes remains a great challenge, and repairing these defects with synthetic bone substitutes is the most common strategy. In general, tissue engineering materials that mimic the structural, mechanical and biological properties of natural bone have been extensively applied to fill bone defects and promote in situ bone regeneration. Hydrogels with extracellular matrix (ECM)-like properties are common tissue engineering materials, among which methacrylate-based gelatin (GelMA) hydrogels are widely used because of their tunable mechanical properties, excellent photocrosslinking capability and good biocompatibility. Owing to their lack of osteogenic activity, however, GelMA hydrogels are combined with other types of materials with osteogenic activities to improve the osteogenic capability of the current composites. There are three main aspects to consider when enhancing the bone regenerative performance of composite materials: osteoconductivity, vascularization and osteoinduction. Bioceramics, bioglass, biomimetic scaffolds, inorganic ions, bionic periosteum, growth factors and two-dimensional (2D) nanomaterials have been applied in various combinations to achieve enhanced osteogenic and bone regeneration activities. Three-dimensional (3D)-bioprinted scaffolds are a popular research topic in bone tissue engineering (BTE), and printed and customized scaffolds are suitable for restoring large irregular bone defects due to their shape and structural tunability, enhanced mechanical properties, and good biocompatibility. Herein, the recent progress in research on GelMA-based composite hydrogel scaffolds as multifunctional platforms for restoring critical bone defects in plastic or orthopedic clinics is systematically reviewed and summarized. These strategies pave the way for the design of biomimetic bone substitutes for effective bone reconstruction with good biosafety.

**Graphical Abstract:**

This review provides novel insights into the development and current trends of research on GelMA-based hydrogels as effective bone tissue engineering (BTE) scaffolds for correcting bone defects, and these contents are summarized and emphasized from various perspectives (osteoconductivity, vascularization, osteoinduction and 3D-bioprinting). In addition, advantages and deficiencies of GelMA-based bone substitutes used for bone regeneration are put forward, and corresponding improvement measures are presented prior to their clinical application in near future (created with BioRender.com).

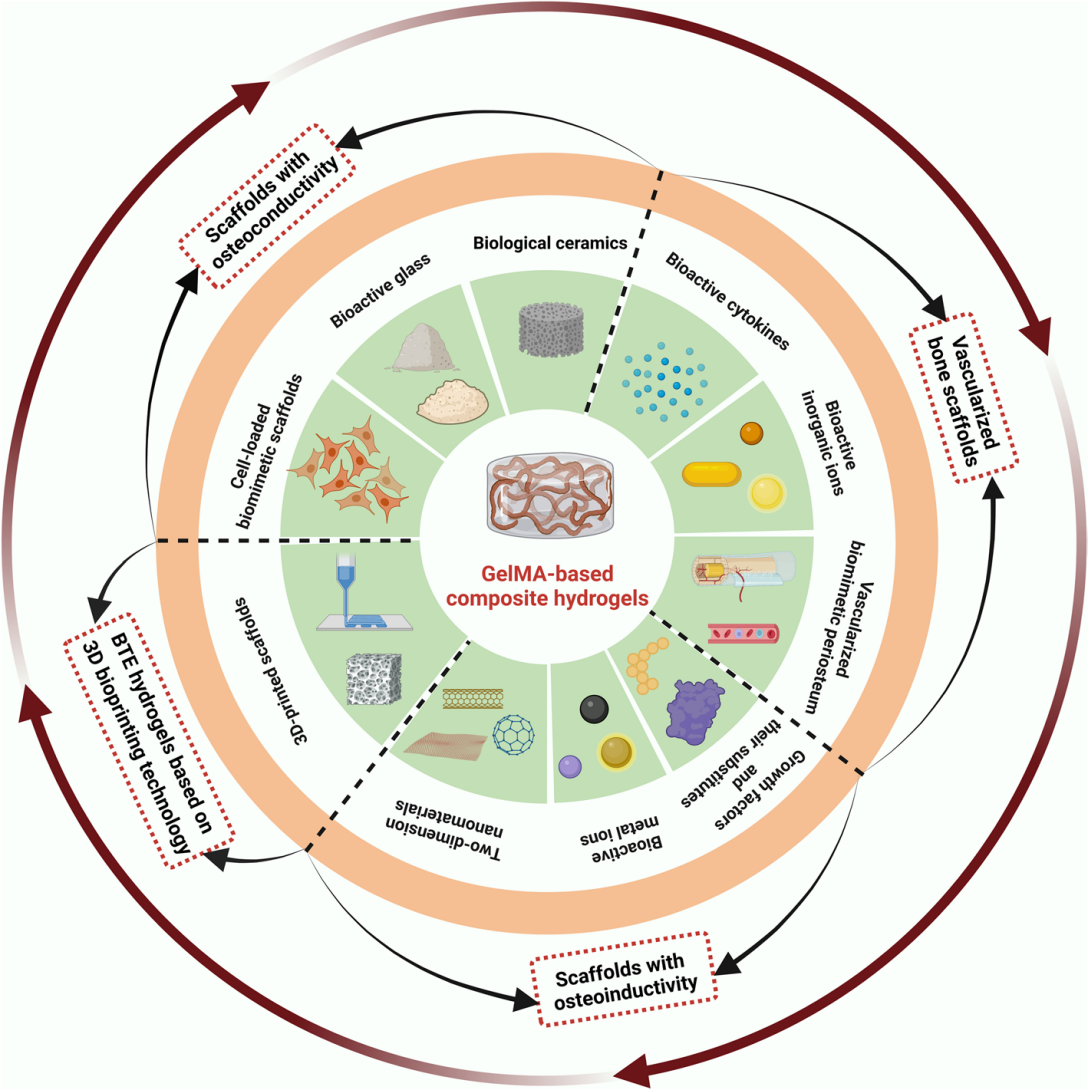

## Introduction

Clinically, the restoration of bone defects resulting from various of pathological conditions, such as severe trauma, tumor resection, infection, degenerative diseases, etc., has been a major challenge for current surgical treatment, which also mean great economical burden for relevant patients [1]. Bone tissue has a certain ability to regenerate; nevertheless, for larger bone defects that are beyond the self-healing capability of bone tissue (often called critical bone defects), bone graft implantation is usually required to achieve effective therapeutic outcomes [2]. The bone grafts commonly used in clinical practice are mainly autologous and allogeneic bone grafts, but such grafts have several potential risks, such as limited sources, donor damage, immune rejection, and possible infection. Therefore, synthetic bone substitutes are being introduced. New bone grafts must not only fill the bone defect area but also promote bone regeneration and repair the normal physiological function of the damaged area. Consequently, the utilization of novel tissue engineering biomaterials that mimic the structural, mechanical and biological properties of natural bone is expected to produce better clinical outcomes for patients with bone defects, decreasing the suffering and economic burden of these patients in clinical practice [3, 4].

The healing process of bone tissues depends on three main aspects: osteoconduction, blood supply and osteoinduction [3, 5, 6]. Bone defect repair is divided into two main categories: primary bone healing, in which a fracture site less than 0.1 mm is firmly stabilized and the bone gap is filled directly by continuous ossification and subsequent Haversian remodeling; and secondary bone healing, which occurs more commonly when the fracture margin is less than half the diameter of the injured bone and involves multiple events, such as blood coagulation, inflammatory response, fibrous cartilage healing tissue formation, intramembranous and endochondral ossification, and bone remodeling. Nevertheless, in some extreme cases of bone healing, such as the healing of large segmental bone defects or critical size bone defects that exceed approximately half the diameter of injured bone tissues, extensive bone loss directly affects revascularization as well as tissue differentiation, ultimately leading to spontaneous fracture and the subsequent development of bone discontinuity without intervention [7, 8]. The function of bone substitutes is primarily a combination of mechanical support and bone regeneration, involving several critical biological properties, such as osteoconductivity, osteoinduction, osteogenesis and osteointegration. Osteoconduction is the capability to facilitate the adherence of osteoblasts and osteogenic progenitor cells and allow these cells to migrate and grow inward within the three-dimensional structure of the graft. Osteoinduction refers to the ability of the graft to induce primitive, undifferentiated and pluripotent cells to develop into a spectrum of osteogenic cells, thereby inducing osteogenesis. Osteogenesis is defined as the osteogenic differentiation and subsequent new bone formation of donor cells from the host or graft. Osseointegration, defined as the anchoring ability of the implant, involves bone tissue formation within the surrounding area of the implant at the bone-implant interface without obvious formation of fiber and other connective tissues [4, 5].

As a common synthetic bone repair material, hydrogels have been extensively utilized to construct bone repair material systems due to their ECM-like properties. In addition, polymer-based bone substitutes that possess suitable physicochemical and bioactive properties exhibit excellent application prospects in BTE. Among them, GelMA, a representative hydrogel formulation, has been extensively utilized in various biomedical fields [9, 10]. It is a dual-bond-modified gelatin that can be crosslinked and solidified into a gel by ultraviolet (UV) or visible light irradiation under the action of photoinitiators, and the scaffolds after gelation possess characteristics of both natural and synthetic biomaterials [10]. The initiation of chain-growth polymerization was triggered by generation of free radicals via homolytic cleavage, and such an unique photo polymerization offers a number of advantages including good injectability, rapid gelation, promoted mechanical properties and bioprinting suitability [9]. More importantly, GelMA hydrogels contain the common Arg-Gly-Asp (RGD) moiety, a tripeptide that facilitates certain important cellular behaviors, including adherence, spreading and differentiation into numerous cell lineages. Moreover, they contain matrix metalloproteinase (MMP) sequences belonging to endopeptidases that facilitate enzymatic degradation, which plays a critical role in tissue rehabilitation and wound closure [11, 12]. In addition, GelMA itself can replace artificial basement membranes or other natural collagen hydrogels because its three-dimensional structure is suitable for cell growth and differentiation, as well as because of its excellent biocompatibility, low antigenicity and cellular response properties [11]. GelMA has also been introduced into bone repair material systems by many researchers because of its good temperature-sensitive gel properties, degradability, adjustable mechanical properties, and ability to promote bone differentiation and vascularization [13, 14]; therefore, a systematic review of GelMA-based construction strategies and recent advances is of great importance to the improvement and development of BTE bone scaffolds.

**Scheme 1:**
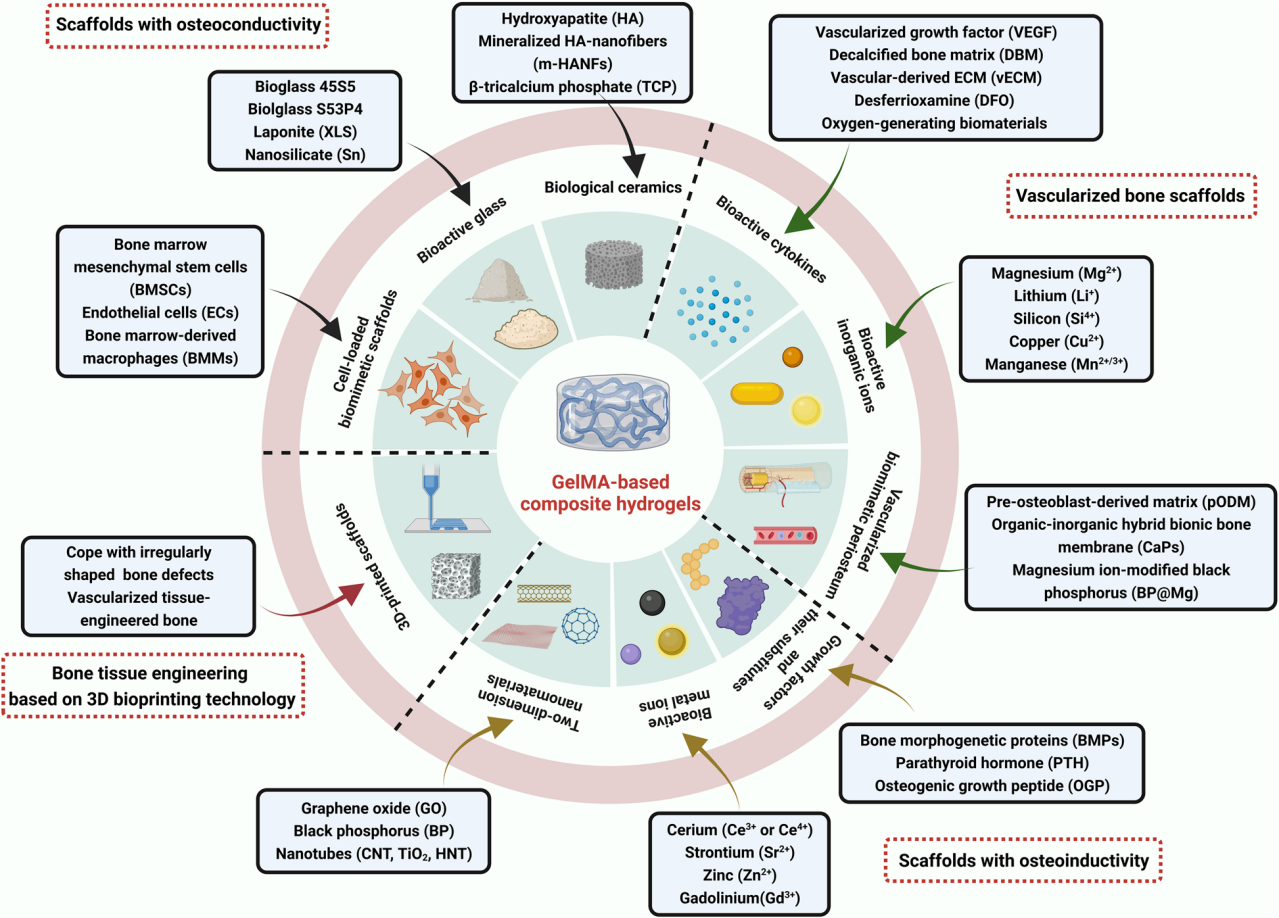
Schematic description of GelMA-based composite hydrogels with multiple functions for bone regeneration (created with BioRender.com)

Ideal bone regeneration scaffolds should exhibit satisfactory cytocompatibility or biocompatibility, biodegradability, excellent bioactivity, suitable biomechanical features and a porous structure to promote cell adherence, proliferation, spreading, nutrients and gas diffusion. However, pure GelMA hydrogels have limited osteogenic potency, and it is necessary to combine GelMA hydrogels with materials with osteogenic activities to improve the osteogenic capability of currently used composites. In addition, the concentration and substitution of GelMA displayed significant influences on the physicochemical properties (porosity, elasticity, compressibility, mechanical stiffness and swelling behavior) and cellular response of pure GelMA hydrogel itself, leading to a certain degree of uncertainty and incapability regarding the regulation of biological behaviours [9]. Moreover, due to weak mechanical strength and deficiency in electrical conductivity of GelMA hydrogels used for developing promising biomaterials in tissue engineering, it is of importance to prepare hybrid hydrogels from mixtures of two or more components to combine the unique superiority of individual properties in the composite hydrogel system [11, 13]. In particular, the poor mechanical rigidity and uncontrollable degradation rate severely limited the wide application of pure GelMA hydrogel scaffold for the treatment of loading burden bone defects, and the combination of GelMA and bioactive ingredients through different strategies resulted in multifunctional BTE scaffolds that outperformed their individual components in terms of mechanical properties, adjustable degradation, osteogenesis capacity, antibacterial effects and antineoplastic activities with the assistance of external stimuli (such as near infrared ray-NIR, magnetic field and ultrasonic wave). Despite the rapid development of bone substitutes designed for bone regeneration, the balance among angiogenesis, osteogenesis, antimicrobial activities, and other indispensable properties adapted to different bone repair microenvironments remains to be better coordinated. Therefore, it is important to clarify the applicability and composition of GelMA-based bone repair implants to accelerate their commercialization and clinical application. Considering the outstanding application advantages and prospects of GelMA hydrogel composite systems in tissue therapeutics and corresponding ideas for their functional improvements, in the present review, the progress and current trends of research on GelMA-based hydrogels as effective BTE scaffolds for correcting bone defects are summarized and highlighted from various perspectives, as shown in Scheme 1, and typical GelMA-based hydrogels with various bioactive properties were also summarized in Table 1.

**Table 1:** Summary of typical GelMA-based hydrogel scaffolds with various bioactive properties for accelerated bone regeneration

Categories	Components	Composite hydrogel scaffolds	Used cells lines	Cell seeding (2D or 3D)	Physical properties	Bioactivities	References	Year of publication
GelMA-based bone scaffolds with osteoconductivity	Biological ceramics	GelMA-HAp-HAD/Col I	rBMSCs	2D	Excellent swelling properties, mechanical stability and delayed degradation	Promoted the migration and differentiation of BMSCs, improved angiogenesis and bone regeneration	[15]	2022
		GelMA-HAP-Sn	rBMSCs	2D	Injection available, good mechanical, swelling and degradation properties	Promoted survival, proliferation and migration of cells, increased expression of osteogenic markers and matrix mineralization	[16]	2021
		GelMA/nHAP/CSA	MC3T3-E1	2D	Improved material stability and compression property, favorable swelling property and degradation ability	Enhanced migration and osteogenic differentiation of osteoblasts, improved bone regeneration	[17]	2023
		GelMA-HAMA/nHAP/USCEXOs	rBMSCs, EPCs	2D	Satisfactory controlled release and suitable biomechanical properties	Promoted angiogenesis and osteogenesis, significantly accelerated bone repair	[18]	2023
		GelMA/m-HANFs	MC3T3-E1, rBMSCs	2D	Improved mechanical, swelling and degradation properties	Biocompatibility, accelerated bone regeneration	[19]	2022
		GelMA/PCL/ β-TCP	rBMSCs, HUVEC, RAW264.7 Schwann cells	2D	Porous structural and good mechanical support function	Biocompatibility, promoted tissue regeneration and reconstruction by improving blood vessel, improved bone remodeling	[20]	2022
		GelMA/β-TCP/Alginate/MXene	rBMSCs, RAW264.7	3D	Good shear thinning properties and suitable viscosity, improved mechanical strength	Biocompatibility, excellent antibacterial properties, promoted healing of infected bone defects and bone regeneration	[21]	2022
	Bioactive glass	BG-XLS/GelMA-DFO	MC3T3-E1, ADSCs	2D	Suitable pore structure and degradability, improved mechanical properties	Promoted osteogenic differentiation of ADSCs, increased expression of HIF-1α and VEGF on ADSCs, promoted regeneration of bone defects	[22]	2021
		BG-GelMA	mBMSCs	2D	Increased compression modulus and mineralization, good swelling behavior and degradation rate	Promoted cell adhesion, proliferation and osteogenic differentiation	[23]	2018
	Biomimetic composite hydrogel	GelMA-BMSCs	BMSCs	3D	Injectability and porous structure	Good cytocompatibility and proliferative properties, promoted new bone formation and angiogenesis	[24]	2021
		GelMA-RF	KUSA-A1	3D	Delayed photocrosslink curing and suitable mechanical function	Increased cell viability, promoted differentiation and maturation of osteoblasts	[25]	2021
		GelMA-BMSCs/PLA-PGA-PLA-ECs	BMSCs, RAOECs	3D	Sufficient mechanical properties and good permeability	Increased cell viability, promoted differentiation and maturation of osteoblasts	[26]	2022
		GelMA/HAMA/Alginate/GO	BMSCs, BMMs	3D	Stable porous structure, suitable mechanical, swelling and degradation properties	Promoted polarization of BMMS to M2 type, promoted osteogenic differentiation of BMSCs, improved osteogenic repair	[27]	2022
GelMA-based vascularized bone scaffolds	Bioactive cytokines	GelMA/HAMA/DBM/VEGF	BMSCs	3D	High mechanical strength, appropriate biodegradation rate and controllable VEGF release	Biocompatibility, excellent ectopic bone regeneration ability, successful repair of a 15 mm long tibial defect in a rabbit	[28]	2021
		Eth-DFO@GelMA/GGMA	BMSCs, HUVECs	3D	Stable grid-like structure, improved printability and mechanical property, slow release of DFO	Promoted migration and tube formation of ECs, improved osteogenesis and angiogenesis	[29]	2022
		vECM-GelMA	BMSCs, HUVECs	2D	High BMP-2 loading efficiency, slow release of BMP-2	Promoted formation and vascularization of new bone	[30]	2022
		CPP-L/GelMA	MC3T3-E1 RAW264.7 HUVECs	2D	Durative release of internal functional components, suitable mechanical function	Promoted angiogenesis and osteogenesis, ROS scavenging ability, inhibit osteoclast differentiation	[31]	2023
		GelMA/OMP	hMSCs	3D	Enhanced mechanical properties, prolonged oxygen release	Good cytocompatibility, promoted osteogenic differentiation and angiogenesis	[32]	2023
	Bioactive inorganic ions	GelMA-BP^a^-Mg	rBMSCs, HUVECs	3D	Injection available, slow release of BP and Mg^2+^	Good tube formation, high expression of e-NOS and VEGF, reduction of TRAP-positive multinucleated osteoclasts, improved osteogenesis and angiogenesis	[33]	2021
		GelMA/Li-MBG	BMSCs, RAW264.7 HUVECs	2D	Good mechanical properties, continuous Li^+^ release	Promoted cell proliferation, angiogenesis, osteogenesis and regulation of macrophages in a high-glucose microenvironment, reduction of M1 macrophages	[34]	2022
		GelMA-PEGDA/SiPAC	BMSCs, HUVECs	2D	Good biodegradability, slow release of silicon and phosphorus ions	Satisfactory biocompatibility promoted bone regeneration and vascularization	[35]	2022
		DFO/MnCO@GelMA	RAW264.7 HUVECs MSCs	2D	Good surface hydrophilicity and mechanical support, the stent is responsive to H_2_O_2_ and continuously releases CO	Good cytocompatibility, reduced M1 polarization of the macrophages, improved angiogenesis and osteogenesis	[36]	2022
		GelMA/GeP@Cu	BMSCs HUVECs NSCs, PC12	2D	Enhanced conductivity, good mechanical properties and suitable swelling behavior	Good antibacterial properties, promoted osteogenic and angiogenic properties, stimulate neurite growth and neural differentiation	[37]	2023
	Vascularized biomimetic periosteum	pODM/GelMA	BMSCs MC3T3-E1	2D	Good adhesion and proliferation properties	Concentration-dependent chemotaxis towards BMSCs, promoted bone repair of segmental bone defects in rabbit radius	[38]	2020
		CaPs@GelMA-F	MC3T3-E1, HUVECs	2D	Good mechanical properties, continuous release of calcium ions	Good biocompatibility, promoted mineralization, osteogenesis and angiogenesis	[39]	2020
		GelMA-BP^b^@Mg/GelMA-PEG-β-TCP	BMSCs, HUVECs, NSCs, PC12	2D	Suitable mechanical properties and swelling behavior	Induction of angiogenesis and peripheral nerve repair, promoted bone regeneration	[40]	2022
GelMA-based bone scaffolds with osteoinductivity	Growth factors and their substitutes	GelMA/BMPs	BMSCs, RAW264.7	3D	Enhanced mechanical properties, sustained release of BMP-4	Biocompatibility, significant increase in BMP-2 expression, induced M2 macrophage polarization and improved inflammatory microenvironment, accelerated bone repair	[41]	2020
		GelMA/PTHrp	MC3T3-E1	2D	Effective in prolonging the release of abaloparatide	Promoted viability, differentiation and mineralization of preosteoblasts, accelerated bone regeneration	[42]	2019
		GelMA/OGP	MC3T3-E1	2D	Slow and sustained release of OGP, good mechanical properties	Promoted bone regeneration	[43]	2020
		GelMA-KP/QK	BMSCs, HUVECs	2D	Self-healing and injectable properties, slow release of KP/QK	Improved osteogenesis and angiogenesis	[44]	2022
	Bioactive metal ions	GelMA/ZIF-8	rBMSCs	2D	Suitable mechanical properties and swelling behavior, continuous release of Zn^2+^	Good cytocompatibility, significantly enhanced expression level of ALP, effective antimicrobial activity, improved alveolar bone regeneration	[45]	2022
		GelMA/Sr-MBGNs	TIID BMSCs Raw 264.7	2D	Enhanced mechanical properties and mineralization, suitable swelling and degradation properties	Increased the level of OCN (NCPs), regulated alignment of hyaluronan on intralaminar mineralization and promotes osteoblast differentiation via Kindlin-2/PTH1R/ OCN axis	[46]	2023
		Ce@GelMA	rBMSCs	2D	Significantly enhanced mechanical properties, rapid capture of detrimental ROS	Good cytocompatibility, promoted bone regeneration	[47]	2022
		Gd-MoS_2_-NAGA/GelMA	ROBs	2D	Excellent photothermal ability, slow release of Gd^3+^	Good cytocompatibility, excellent antimicrobial and antitumor properties, promoted new bone formation	[48]	2023
	Two-dimensional nanomaterials	GelMA/SiGO	hMSCs	3D	Enhanced production, retention and bioactivity of BMPs	Improved mineralization and accelerated bone repair	[49]	2021
		GelMA/HAMA/Alginate/GO	BMSCs, BMMs	3D	Stable porous structure, suitable mechanical, swelling and degradation properties	Promoted polarization of BMMs to M2 type, promoted osteogenic differentiation of BMSCs, improved osteogenic repair	[27]	2022
		GelMA/BP^b^@Mg	BMSCs, SCs, PC12	2D	Improved mechanical properties, suitable photothermal properties	High antibacterial activity, improved local inflammatory microenvironment, promoted regeneration of bone and CGRP nerve fibers	[50]	2023
		GelMA/CNT	NIH-3T3, hMSCs	3D	Improved mechanical properties	Good cytocompatibility	[51]	2012
		GelMA/TiO_2_	hBMSCs, BMMs, RAW 264.7	2D	Well preserved nanotubular morphology, slow release of Mg^2+^	Exhibited favorable effects on growth rate and bone formation capacity	[52]	2019
		GelMA/HNT	hDPSCs	2D	Good mechanical, swelling and degradation properties	Good cytocompatibility, accelerated bone formation	[53]	2019
3D-bioprinted GelMA-based bone scaffolds		GelMA/β-TCP/Alginate/MXene	rBMSCs, RAW264.7	3D	Good shear thinning properties and suitable viscosity, improved mechanical strength,	Biocompatibility, excellent antibacterial properties, promoted healing of infected bone defects and bone regeneration	[21]	2022
		GelMA-BMSCs/PLA-PGA-PLA-ECs	BMSCs, RAOECs	3D	Sufficient mechanical properties and good permeability	Exhibited a coupling effect between angiogenesis and osteogenesis, in situ vascularization, effectively promoted new bone formation	[26]	2022
		GelMA-Alg-HUVECs/ GelMA-Alg-WH/HAP-hMSCs	HUVECs, hMSCs	3D	Good mechanical, swelling, degradability properties	Good cytocompatibility and excellent osteogenic properties	[54]	2023
		GelMA-PEGDA/SiPAC	BMSCs, HUVECs	2D	Good biodegradability, slow release of silicon and phosphorus ions	Satisfactory biocompatibility, promoted bone regeneration and vascularization	[35]	2022
		Eth-DFO@GelMA/GGMA	BMSCs, HUVECs	3D	Stable grid-like structure, improved printability and mechanical property, slow release of DFO	Promoted migration and tube formation of ECs, improved osteogenesis and angiogenesis	[29]	2022
		GelMA/HA-Ce	MC3T3-E1	3D	Significantly enhanced mechanical properties, exhibited a uniformly porous microstructure	Good cytocompatibility, promoted bone regeneration	[55]	2022
		GelMA-BMSCs/Dextran emulsion	rBMSCs	3D	Porous structure, good mechanical and degradation properties	Promoted cell proliferation, migration, spreading and osteogenic differentiation of rBMSCs via regulation of YAP signal pathway, improved bone healing	[56]	2022
		GelMA/BPs	Cells from the BPs	3D	Strong shear thinning behavior and high gel strength	Improved osteogenesis	[57]	2020
		GelMA/Alg/C3S	hADSCs	2D	Good printability, improved mechanical properties	Satisfactory cytocompatibility and osteogenic capacity	[58]	2023
		GelMA/CQDs	hBMSCs, hECs RAW264.7	2D	Excellent printability and photothermal properties	Anti-inflammatory activity, promoted osteogenic and angiogenesis, NIR-triggered anti-osteosarcoma performance and vascularized bone regeneration	[59]	2023
		GelMA-PPy-Fe	hBMSCs RAW264.7	3D	Excellent shape fidelity, enhanced conductivity	Good cytocompatibility and improved osteogenic differentiation	[60]	2023

## GelMA-based bone scaffolds with osteoconductivity

**Fig. 1:**
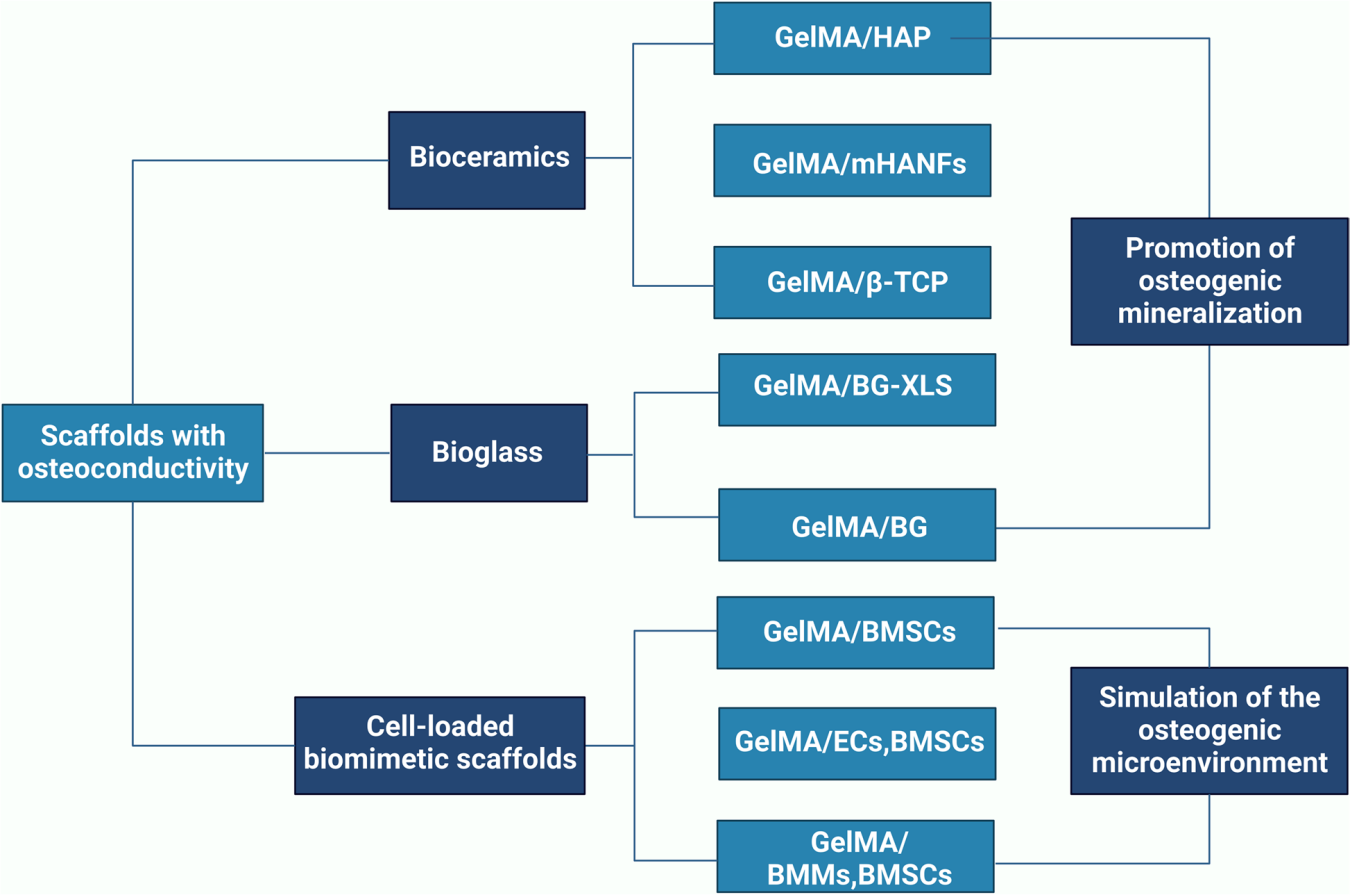
Schematic description of GelMA-based bone scaffolds with osteoconductivity (created with BioRender.com)

Severe fractures or large bone tissue defects attributed to trauma, infection, tumors or other diseases are usually unable to heal by bone tissue self-repair, and the bone shape and function must be restored by bionic reconstruction techniques, which often require the use of BTE materials [4]. An ideal candidate for effective bone regeneration is expected to have optimal porosity, biomechanical strength, physicochemical properties, biodegradability, biocompatibility and osteoconductivity and to serve as a transfer station for the unhindered delivery of nutrients, waste and gas exchange of anchored cells within the scaffolds. Osteoconductivity usually refers to the ability of implanted biomaterials that support new bone tissues formation on the materials surface, which depends on the bone repair conditions and cellular reactions toward the biomaterials used [5]. To improve the osteoconductivity of BTE scaffolds, inorganic materials (such as calcium phosphate ceramics and bioactive glass) are most commonly used, as well as cell-loaded bionic scaffolds are summarized in this subsetion. More importantly, the incorporation of these components mentioned above provides the necessary biomechanical properties and bioactivity to support bone regeneration in different load-bearing applications and to facilitate bone-biomaterial integration (Fig. 1).

### Biological ceramic-incorporated composite hydrogels

Bioceramics mostly refer to calcium phosphate ceramics, including hydroxyapatite (HAP) or β-tricalcium phosphate (β-TCP), which are composed of several inorganic components, forming a mineral phase with a chemical composition similar to that of calcified tissue, as a synthetic mineral salt. Due to their excellent osteoconductivity and resorbability, these materials are often used in clinical studies for bone defect repair [4, 61]. GelMA hydrogels can serve as excellent carriers for these inorganic materials to prepare bionic bone scaffolds with the assistance of 3D-printing and microfluidic technologies, which can improve the osteoconductivity of the bone scaffolds while also affecting the interactions between the scaffolds and the tissues at the site of bone defects to accelerate the bone repair process. HAP is the main constituent part of the bone inorganic matrix, accounting for approximately half of the bone weight, and has been widely used in constructing bone substitutes for bone defect repair. However, its development for use in BTE has always been limited by the disadvantages of difficult molding and mechanical brittleness. In addition, the osteogenic ability of HAP or β-TCP is insufficient, and bioceramics alone do not show satisfactory osteogenic ability [13]. In view of this dilemma, researchers have made numerous efforts to optimize the osteogenic activity and osteoinductive ability of bioceramics by introducing various composite materials, such as ECM-like hydrogels and nanosilicates, that could improve the structure of HAP or β-TCP to promote bone regeneration.

#### GelMA/HAP-based hydrogels

HAP one of the most common classes of calcium phosphate ceramic materials, is widely used in bone tissue engineering [3]. In one study, Pu et al. used a 3D printed GelMA-HAP scaffold as the main body and introduced a biohybrid hydrogel as an ECM component to form a biomimetic bone implant material (HGH), which was referred to as HAD/Col I. The amide reaction of the amino group of dopamine with the side chain carboxyl group of hyaluronic acid (HA) was applied to produce dopamine-modified HA (HAD) compounds [15]. The results showed that dopamine-modified polysaccharide hybrid hydrogels based on collagen I (Col I) and HA derivatives facilitated the proliferation and spreading of rabbit bone marrow mesenchymal stem cells (rBMSCs) and enhanced the in vitro enrichment and migration of rBMSCs through potential functional protein adsorption [62]. In this study, 3D-printed GelMA/HAP (GH) scaffolds and hydrogel-anchored 3DP GelMA/HAP (HGH) scaffolds were prepared. Characterizing and comparing the two revealed that the HGH scaffolds outperformed the GH scaffolds in terms of the swelling rate, elastic modulus, and thermogravimetric weight and exhibited excellent water retention, mechanical stability, and delayed degradation. In accordance with the results of in vitro experiments, the HGH scaffolds promoted the migration, proliferation and osteogenic differentiation of bone marrow stem cells, and the rabbit cranial defects treated with the HGH scaffolds demonstrated accelerated new bone formation, verifying the in vivo therapeutic of this composite scaffold with osteoconductive property.

The osteoconductive ability of pure GelMA-HAP hydrogels is still insufficient for highly effective bone regeneration due to the limited osteogenic ability of HAP. Nanosilicate (Sn) [Na^+^_0.7_[(Si_8_Mg_5.5_:Li_0.3_)O_20_(OH)_4_]^−^_0.7_)] is a charged synthetic bioactive silicate nanosheet (20–30 nm in diameter and 1 nm in thickness). The magnesium ions released by Sn dissociation could mediate the integration of proteins from the integrin family by inducing the expression of adhesion proteins and promoting the initial adhesion of cells to the biomaterial surface, while the Sn degradation products, protosilicate (Si(OH)_4_) and lithium, could promote the synthesis of an important bone ECM component, type I collagen [63]. It has been previously confirmed that lithium enhances the osteogenic effect of Sn by affecting the expression of runt-related transcription factor 2 (RUNX2), which is the main osteogenic regulator responsible for coordinating the expression of bone-specific genes [64]. Thus, Sn can effectively induce the osteogenic differentiation of mesenchymal stem cells (MSCs) without the addition of any auxiliary exogenous osteoinductive factors. A mimetic injectable GelMA-HAP-Sn BTE material system loaded with MSCs, nanohydroxyapatite (nHAP) and Sn was designed and prepared in another study. The in vitro findings demonstrated that GelMA-HAP-Sn hydrogels promoted increased cell survival, proliferation and migration behavior, with increased expression of osteogenic markers and increased matrix mineralization of bone marrow mesenchymal stem cells (BMSCs). Furthermore, BMSC-coated GelMA-HAP-SN hydrogels were injected into rat critical-sized cranial defects, and micro-computed tomography (micro-CT) and histomorphometric staining results further confirmed their good bone regeneration ability in vivo [16].

**Fig. 2:**
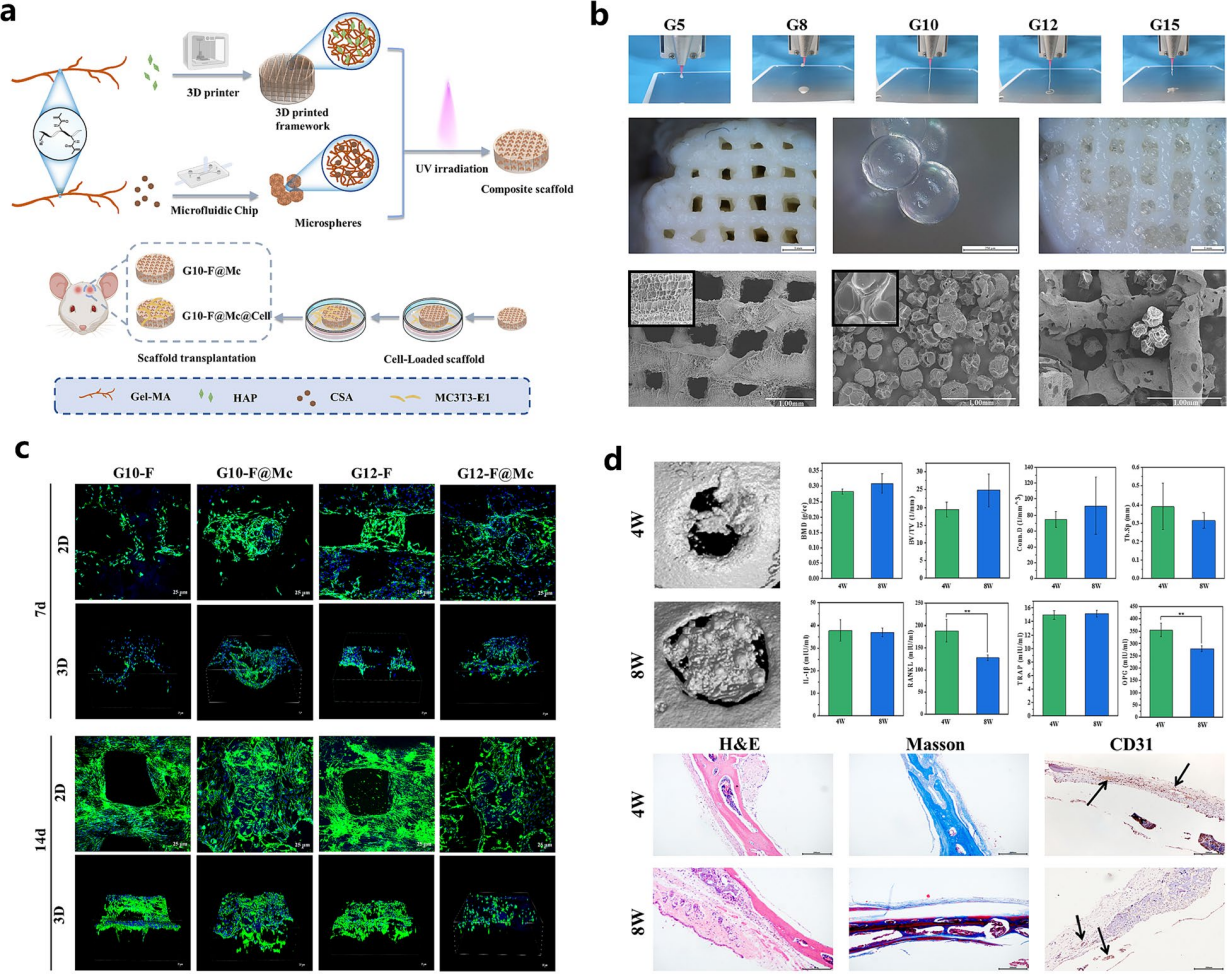
Biocompatible composite hydrogel scaffold integrated with GelMA/nHAP and CSA microspheres conjugated 3D porous frameworks for effective bone repair. **a** Graphic description of the preparation and cell-laden G10-F@Mc hydrogel scaffold for repairing bone defects. **b** Surface characterization of bio-inks and 3D printed frameworks within the composite hydrogel scaffolds. **c** In vitro cell behaviors of MC3T3-E1 cells on the hydrogel scaffolds for 7 and 14 days. **d** In vivo bone regeneration of the hydrogel scaffolds in the mouse skull defects for 4 and 8 weeks. Images reproduced from [17], © 2022 Elsevier Ltd

nHAP, a main inorganic component of the natural bone matrix, exhibited clear biological effects on osteogenesis, osteoconductivity and bone formation, and hybrid scaffolds incorporating different contents of nHAP biofunctionalized with VEGF provided a favorable metabolic microenvironment for effective therapeutic of osteonecrosis due to significant potentiation of angiogenesis and osteoconduction [65]. Meanwhile, the addition of nHAP into the GelMA network significantly improved the biomechanical properties, such as mechanical stiffness and physiological stability, and the biocompatibility of pure GelMA hydrogels [66]. Guo et al. reported a 3D framework composed of GelMA and nHAP, and microspheres made of GelMA and chondroitin sulfate A (CSA) were incorporated into the framework as bridges and channels for cell adherence and migration [17] (Fig. 2). The prepared hydrogel scaffolds demonstrated a uniform porous structure that was conducive to subsequent cell attachment, and the material stability and compression property of the scaffolds were improved after the modification of microspheres. The migration and osteogenic differentiation of osteoblasts were significantly enhanced by the CSA released from the microspheres, and the in vivo bone regenerative capacity of the hydrogels was further validated in a mouse calvarial defect model. These multimodule bioactive hydrogel scaffolds fabricated using 3D printing technology provided a favorable bone tissue microenvironment for the effective interaction of cells and bioactive factors, thus facilitating the healing of bone defects. Inspired by the multifunctional differentiation potential of human urine-derived stem cell exosomes (^USC^EXOs), Lu et al. manufactured a GelMA-methacrylated hyaluronic acid (HAMA)/nHAP composite hydrogel as a delivery platform for the efficient encapsulation and slow release of ^USC^EXOs to achieve better osteogenesis [18]. As expected, satisfactory controlled release and suitable biomechanical properties of the GelMA/nHAP-based hydrogel scaffolds were recorded, and the in vitro osteogenesis of BMSCs and angiogenesis of endothelial progenitor cells (EPCs) were clearly promoted after the administration of the ^USC^EXOs/GelMA-HAMA/nHAP composite hydrogel scaffolds. Moreover, significantly accelerated bone healing was observed in a rat cranial bone defect model implanted with these composite substitutes, indicating the potential of these composite hydrogels as a therapeutic exosome delivery system coupled with the ability to promote osteogenesis and angiogenesis for effective bone regeneration. As mentioned above, the introduction of HAP into the GelMA hydrogel system to enhance the osteoconductive properties of scaffolds is currently a commonly used approach in bone tissue engineering research.

#### m-HANFs/GelMA hydrogel

The bone regeneration ability of hydrogel scaffolds was significantly promoted by adding different inorganic nanoparticles (such as HA, β-TCP and bioactive glass nanoparticles) into hydrogel scaffolds, as previously reported [67, 68]. The results showed that these fillers can modify the hydrogel structure to some extent and improve its mechanical strength and osteoinductivity. However, when these nanoparticle fillers were added at relatively low levels, the improvement in the mechanical strength of the material was limited. Moreover, it is difficult to achieve a uniform dispersion of nanoscale particles in the polymer matrices, especially when the filler addition is increased to relatively high levels because the excess filler tends to agglomerate, which negatively affects the mechanical properties of the resulting substrate and may influence its final osteogenic capability [68]. Consequently, one-dimensional bioceramic fillers, such as HA nanofibers (HANFs), glass fibers or halloysite nanotubes, with high aspect ratios are regarded as more promising reinforcing materials because of their better support and ability to bridge and form networks. A collagen-derived gelatin/HA nanocomposite, a synthetic inorganic/polymer hybrid biomaterial with a chemical composition analogous to that of biological bone tissue, showed improved bioactivity and mechanical properties compared to those of gelatin alone [69].

Despite the improvement of biological activity and mechanical property of GelMA hydrogel due to the addition of HANFs, the degree of crystallization of traditional synthesized HANFs are still differs from those of natural bone tissues in organisms, therefore, it is of great significance to develop biomimetically modified HANFs that are more close to natural bone tissues to promote the bone regenerative capacity of HANFs [69]. One study prepared HA-nanofibrous scaffolds (HANFs) with ultrahigh aspect ratios and mineralized them in simulated body fluids (SBFs) to coat their surfaces with a layer of bone-like apatite [19]. The surface of the mineralized HANFs (m-HANFs) was coated with a layer of trace element-doped biomimetic apatite minerals that mimicked natural bone apatite in terms of structure and composition, which facilitated bone regeneration. Then, different amounts of m-HANFs were added into GelMA to form m-HANF/GelMA composite hydrogels. Increasing the amount of m-HANFs resulted in the formation of a denser network inside the composite hydrogels, which improved their mechanical properties and energy dissipation ability. Notably, there was a significant discrepancy in the in vitro and in vivo biological effects of the HANFs with different incorporated contents, which may be closely related to the homogeneity of the network structure within the prepared GelMA-based hydrogel scaffolds. These results suggested that the addition of m-HANFs can improve the mechanical properties of GelMA and may provide more stable support for bone regeneration [19]. Considering the possible inconsistency between the in vitro cellular experiments and in vivo animal experiments, it is necessary to clarify the key factors contributing to these contradictions prior to real clinical application. In addition, there is no absolute correlation between the incorporated content of inorganic fillers and the bone regenerative capacity of the synthetic hydrogels; thus, the proportion of inorganic mineral substances and GelMA should be further optimized to achieve the expected bone healing outcomes in an established bone defect model.

#### GelMA/β-TCP-based hydrogels

**Fig. 3:**
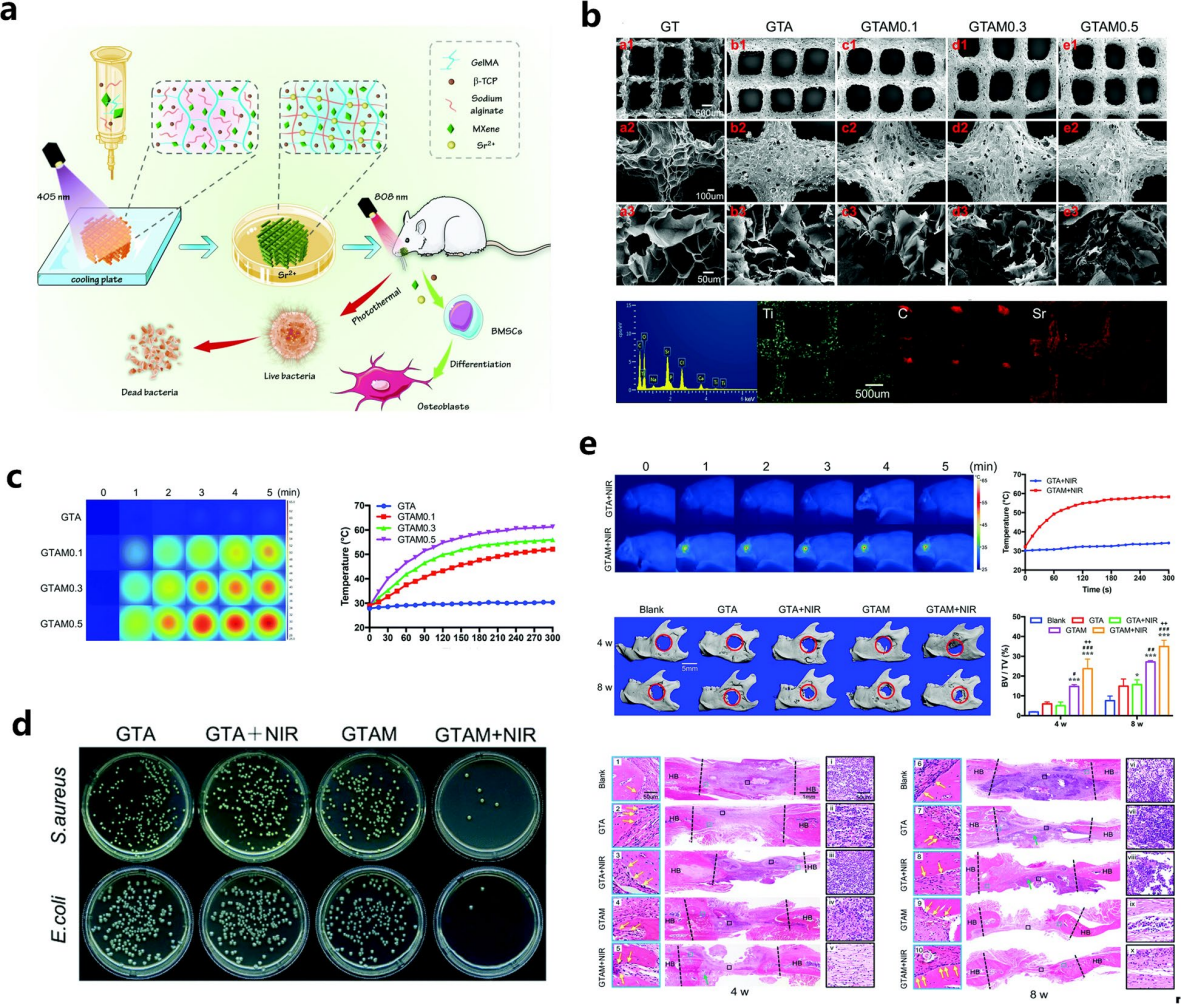
GelMA/β-TCP-based hydrogel scaffold decorated with personalized MXene (Ti_3_C_2_) with excellent photothermal antimicrobial and osteogenic capabilities for the therapy of infected bone defects. **a** Schematic illustration of the fabrication, in vitro biological effects and in vivo bone repair efficacy of the GelMA/β-TCP/Sr^2+^/MXene (GTAM) hydrogel scaffold. **b** Surface characterization of different 3D-printed hydrogel scaffolds. **c** Determination of the NIR-responsive photothermal properties of different 3D-printed scaffolds. **d** Representative images of *S. aureus* and *E. coli* clones cocultured with 3D-printed scaffolds with or without NIR irradiation for 24 h. **e** Determination of the in vivo photothermal effect and bone regenerative actions of the hydrogel scaffolds via radiographic and histological analysis. Images reproduced from [21], © 2022 The Royal Society of Chemistry

In addition to HAP, another typical bioceramic, β-TCP, has been widely used to optimize the biomechanical performance and bioactive potency of GelMA hydrogels. According to the report from Ji et al. a 3D-printed scaffold composed of GelMA, polycaprolactone (PCL) or β-TCP was prepared, and the regulatory effects of this 3D-printed scaffold on the symbiotic microenvironment during bone regeneration were clarified using the ribonucleic acid sequencing (RNA-Seq) method [20]. Cell recruitment and extracellular matrix adherence were promoted in the niche of the scaffolds, which exhibited excellent in vitro biocompatibility. Then, the author established a rat femur critical-sized bone defect model, and the implantation of the hydrogel scaffolds was found to contributed to accelerated bone healing. RNA-seq technology followed by Gene Ontology (GO) and Kyoto Encyclopedia of Genes and Genomes (KEGG) analysis showed that many immune-associated pathways were activated after GO and KEGG analysis, indicating that the hydrogel scaffold itself served as a beneficial matrix with immunoregulation for tissue regeneration. In summary, the transcriptome function analysis provides insight into the interactions between cells and scaffolds at different stages after implantation, and related signaling pathways involved in bone healing and potential adverse inflammatory responses were elucidated, making it possible to prepare effective engineered bone products with more accurate therapeutic potency. In the investigation performed by Nie et al. a GelMA/β-TCP-based hydrogel scaffold decorated with personalized MXene (Ti_3_C_2_) with excellent photothermal antimicrobial and osteogenic capabilities was prepared using 3D printing technology and found to coped appropriately with irregularly shaped infected bone defects [21] (Fig. 3). In this study, a hydrogel mixture of GelMA, β-TCP, and alginate (Sr^2+^) was used as the base material for 3D printing, and MXenes, a novel type of transition metal carbide/nitride/carbon nitride that can kill both gram-positive and gram-negative suspended bacteria and microbial biofilms by destroying bacterial membranes through direct physical contact, were introduced to enhance the antimicrobial properties of this hybrid substitute. The antimicrobial properties of this new transition metal carbide/nitride/carbon-nitride were particularly high under NIR irradiation (808 nm). In this study, rat BMSCs were mixed into GTAM bioink for 3D bioprinting, and the cell-filled 3D-printed GTAM scaffolds showed biocompatibility and bone-forming ability under a high risk of bacterial infection, providing a multifunctional bone scaffold with excellent photothermal conversion efficiency and osteogenic activities for synergistic therapy of infected bone defects. Moreover, Mahmoud et al. prepared a photocrosslinked composite fiber membrane (GelMA/PCL-TCP) using electrostatic spinning technology [70]. Due to the relatively weak mechanical and osteogenic properties of GelMA hydrogels, this study added PCL to enhance its mechanical properties and additionally introduced β-TCP to enhance its osteogenic properties. As it is well-known, the technology of electrostatic spinning can fabricate micro/nanofibers with mechanical and electrical properties, such as larger specific surface area, high porosity, high flexibility, and high electrical conductivity [71]. These results showed that the composite microfibers have a uniform porous structure and good mechanical properties and that they promote the attachment, proliferation, mineralization and osteogenic gene expression of alveolar bone mesenchymal stem cells (ABMSCs) and improve new bone formation. In this study, a GelMA composite hydrogel with good mechanical and osteogenic properties was prepared through the doping of functional materials combined with electrostatic spinning technology, which provides a novel and potential direction for the future application of GelMA hydrogels in bone tissue engineering.

### Bioactive glass-incorporated composite hydrogels

Bioglass (BG), a category of synthetic silicate-based ceramics, originally consisted of silicon dioxide (SiO_2_), sodium oxide (Na_2_O), calcium oxide (CaO), and phosphorus pentoxide (P_2_O_5_) when first developed in the 1970s. For better stability, these ceramics were restructured by the incorporation of potassium oxide (K_2_O), magnesium oxide (MgO), and boron oxide (B_2_O), and the key component, silicate, subsequently accounted for approximately 50% by weight. When exposed to biological fluids, ions including Si, Ca and P are rapidly released from BG and form a hydroxycarbonate apatite (HCA) surface layer. This thin HCA coating absorbs proteins and attracts bone progenitor cells. Furthermore, this bioapatite layer is partially replaced by bone tissues during long-term implantation through a creep replacement process [3]. To summarize, BG 45S5 (46.1 mol.% SiO_2_, 24.4 mol.% Na_2_O, 26.9 mol.% CaO and 2.6 mol.% P_2_O_5_, now sold by NovaBone Products LLC, US) and S53P4 (53.8 mol.% SiO_2_, 22.7 mol.% Na_2_O, 21.8 mol.% CaO and 1.7 mol.% P_2_O_5_, now sold by BonAlive Biomaterials, Finland) are two of the most widely recognized commercial BGs available on the market as bone graft substitutes [72].

#### BG-XLS/GelMA-DFO hydrogel

As one of commonly used bioglass, BG 45S5, has good bioactivity and osteoconductivity along with the ability to bind to living bone tissue. However, the application of BTE 3D BG scaffolds is usually restricted by their inherent brittleness, low fracture toughness and compressive deformation, as well as unsatisfactory osteoinductivity [73]. The mechanical properties of BG scaffolds can, however, be illustrated by doping other metal ions or polymers into silica-based networks. A previous study confirmed that 2D Sn (laponite, XLS), a magnesium silicate (Na^+^_0.7_[(Si_8_Mg_5.5_:Li_0.3_) O_20_(OH)_4_]^−^_0.7_), served as a crosslinker of molecules and significantly improved the mechanical properties of polymer matrices. In addition, XLS was found to promote cell adhesion, proliferation and osteogenic differentiation [74]. Desferoxamine (DFO) is a hypoxia-mimetic agent that promotes bone regeneration by activating hypoxia-inhibitable factor-1α (HIF-1α)-mediated angiogenesis [75]. A novel BG-XLS/GelMA-DFO scaffold was developed in which XLS significantly enhanced the mechanical properties of the scaffold compared to those of a pure BG scaffold without affecting its mineralization and promoted the osteogenic differentiation of human adipose mesenchymal stem cells (ADSCs). The immobilization of DFO-loaded GelMA hydrogels onto XLS-functionalized BG scaffolds achieved sustained release and inhibited DFO degradation, and in vitro data showed increased expression of HIF-1α and vascular endothelial growth factor (VEGF) by ADSCs. In vivo data showed that the BG-XLS/GelMA-DFO scaffold exhibited strong pro-bone healing ability in a rat cranial defect model 8 weeks after implantation [22]. BG has excellent osteoconductive properties but lacks angiogenic and osteoinductive activities, and it is a feasible strategy to compound other biomaterials with pro-angiogenic and osteoinductive abilities in BTE substitutes.

#### BG/GelMA hydrogel

In one study conducted by Zheng et al. photocrosslinkable bionic BG/GelMA composite hydrogels were prepared by sequential physical and chemical crosslinking (gel + UV) methods [23]. Briefly, different amounts of BG were dispersed into the GelMA solutions, followed by physical crosslinking. Immediately after incubation, "enhanced" composite hydrogels were obtained by photocrosslinking. The four sets of hydrogels had highly interconnected porous structures, with BG uniformly distributed in the composite hydrogel network. In addition, in terms of mechanical properties, the "enhanced" composite hydrogels had a higher compressive modulus than the "conventional" composite hydrogels. This study showed that the mechanical properties and cellular behaviors of the hydrogel scaffolds significantly improved after the addition of BG due to the reliable interactions between the GelMA polymers and BG powder. The “enhanced” composite hydrogels showed good mine good mineralization capacity, and the in vitro results showed that the BG/GelMA composite hydrogels facilitated cell attachment, proliferation and osteogenic differentiation, combined with the interesting crosslinking method for GelMA, signifying their promising application in the development of biomaterials for promoting bone regeneration.

### Cell-loaded biomimetic composite hydrogel

BTE-associated biomaterials capable of mimicking the structural, mechanical and biological properties of natural bone, i.e., bionic scaffolds, are currently a popular research topic. Scaffolds and cells are the basic components of BTE, and correctly combining these two materials while satisfying the requirements of mechanical properties as well as biological activity is currently the most common BTE method. GelMA hydrogels, as a class of hydrophilic polymers with three-dimensional structures, good biocompatibility, biodegradability and weak immunogenicity, have been applied in various studies and exhibit advantageous abilities to promote cell adhesion and proliferation, making them a very good platform for cell loading [9, 10]. BMSCs are the most commonly used stem cells in cell therapy and tissue engineering due to their ability to mobilize and migrate from bone marrow to damaged tissues to repair bone and cartilage defects [76]. GelMA hydrogels lack the osteogenic induction capacity required for bone mineralization and are often used in various studies in combination with stem cells, such as BMSCs or osteoblasts, to prepare cell-loaded scaffolds to enhance the bioactivity and bone repair capacity of hydrogel materials [24, 25].

Li et al*.* fabricated an injectable GelMA hydrogel loaded with BMSCs [24]. In this study, BMSCs were mixed with GelMA solution to which a photoinitiator was added, followed by in situ injection at bone defect sites and then by crosslinking and molding under UV irradiation. The BMSC-loaded hydrogels prepared in the in vitro cellular experiments exhibited good cytocompatibility as well as proliferative properties; the BMSC group and the BMSC-loaded hydrogel group showed vigorous bone growth, new blood vessels and more newly formed bone tissues with mature tissue structure in the bone defect area. The free radical polymerization of GelMA hydrogels is usually initiated by exposure to UV light with the assistance of photoinitiators. Given the damaging effects of UV light on cells and tissues, Goto et al*.* used riboflavin (RF) as a photosensitizer for GelMA hydrogel polymerization under visible light, providing a safer and more effective environment for loaded cells [25]. The GelMA-Irgacure2959 (IR) hydrogel had a similar to that of the UV light-irradiated GelMA-Irgacure2959 (IR) hydrogel, except that the visible light-irradiated GelMA-RF hydrogel required a longer time to polymerize. In vitro experiments showed that KUSA-A1 cells encapsulated in GelMA hydrogels polymerized with visible light had significantly higher viability than those encapsulated in GelMA hydrogels. In terms of osteogenic activity, the late bone formation marker osteocalcin (OCN) was clearly expressed in the KUSA-A1 cells encapsulated in the GelMA-RF hydrogels, whereas the levels of the early markers RUNX2 and osterix (OSX) were downregulated. Additionally, KUSA-A1 cells aggregated and exhibited spherical structures when cultured in the GelMA-RF hydrogels, indicating that the cells cultured in the 3D environment were in a later stage of differentiation, and the 3D matrix structure of the GelMA-RF hydrogels led to high levels of osteoblast differentiation and maturation, indicating the suitability of GelMA-RF hydrogel cultures for osteoblast osteogenesis in vitro.

**Fig. 4:**
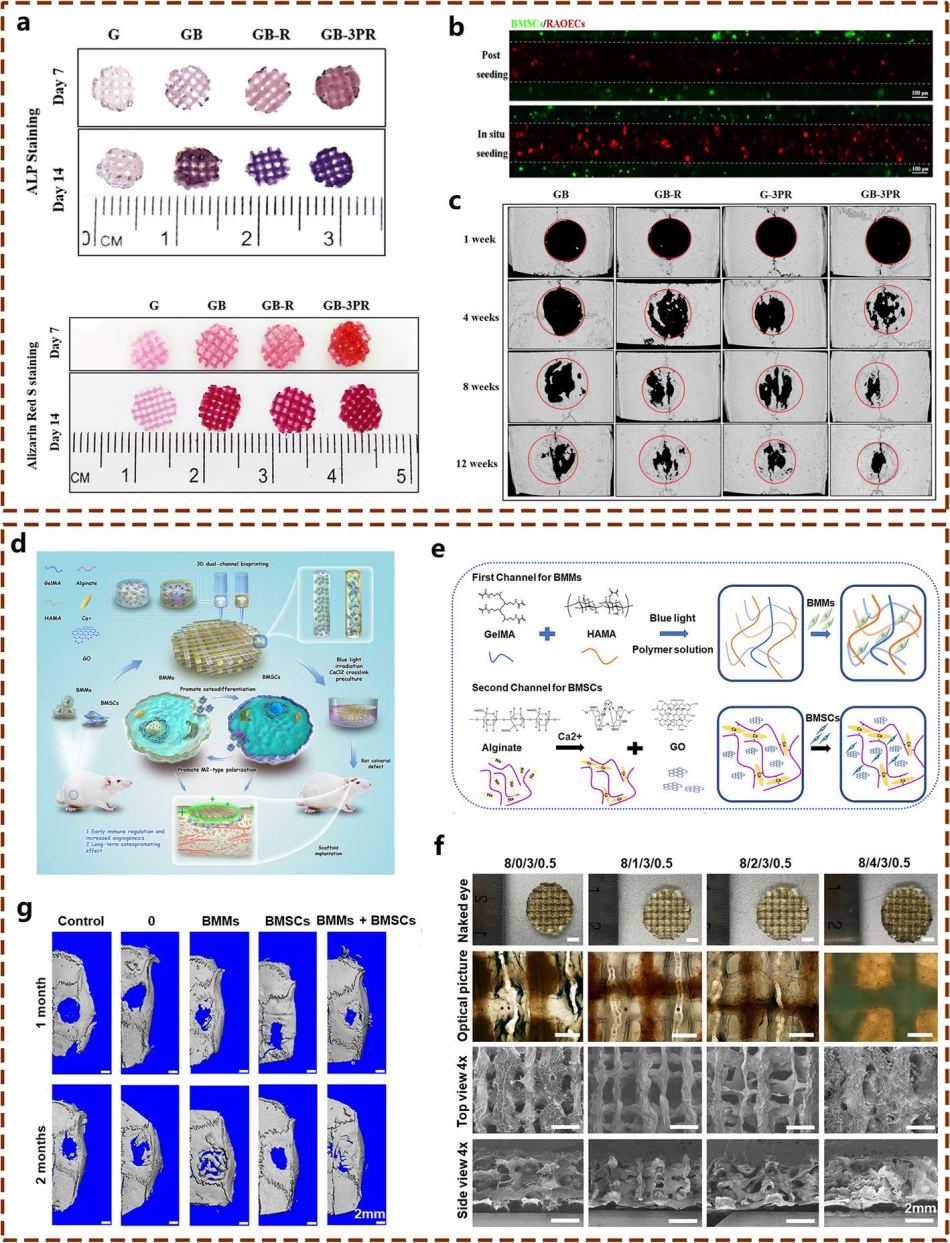
Cell-laden 3D-bioprinted tissue engineered bone substitutes with excellent osteogenic potential for repairing bone defects. **a** In vitro osteogenic performances of GelMA hydrogel scaffold loaded with BMSCs and ECs prepared by 3D-bioprinting technology. **b** CLSM observation of the in situ 3D seeding of BMSCs and RAOECs. **c** In vivo bone repair effect of the cell-loaded GelMA hydrogel on a rat critical-sized calvarial defect model as confirmed by micro-CT evaluation. Images reproduced from [26], © 2022 Elsevier© 2022 American Chemical Society. **d** Graphic description of the 3D bioprinting, in vitro and in vivo experimental procedures used to test the hybrid hydrogel composed of GelMA/HAMA, alginate, GO, rBMMs, and rBMSCs. **e** Graphic illustration of the bioink composition in two channels. **f** Morphological characterization of different 3D bioprinted scaffolds. **g** In vivo bone regeneration of rat calvarial defects implanted with different hydrogel scaffolds. Images reproduced from [27], © 2022 American Chemical Society

In addition to being loaded with BMSCs to enhance its osteogenic activity, GelMA can be loaded with other cell types, such as endothelial cells (ECs) to promote bone tissue angiogenesis and vascularization or bone marrow-derived macrophages (BMMs) to inhibit inflammatory responses and promote osteogenic repair to further promote bone defect repair. One study constructed in situ vascularized tissue-engineered bone using 3D-bioprinting technology [26] (Fig. 4a-c), with GelMA as the matrix bioink, uniformly inoculating ECs and BMSCs on the porous scaffold surface to form a scaffold with effective angiogenic and osteoinductive activity for bone defect restoration. As demonstrated in the in vitro results, a visible coupling effect between angiogenesis and osteogenesis was found in this in situ vascularized scaffold. In vivo investigation further confirmed that the scaffold promoted osteogenic repair. Importantly, amelioration of the inflammatory microenvironment is also a crucial aspect of effective bone repair. Considering the important role of the host immune response to implanted bioengineered bone substitutes, Yu et al. used 3D bioprinting technology to introduce BMMs into a scaffold integrated with GelMA and HAMA hydrogels as an encapsulation system [27] (Fig. 4d-f), with the introduction of BMSCs to further promote the osteogenic activity of the synthetic scaffold. The results showed that BMSCs could promote the polarization of BMMs to the M2 type, decrease the expression of proinflammatory genes and increase the expression of anti-inflammatory genes in the early stage, while BMMs could promote BMSC osteogenic differentiation and further promote osteogenic repair. This dual-channel system resulted in effective bone repair in a rat calvarial defect model by early immune regulation and late osteogenesis induction. This investigation reported the 3D multichannel bioprinting of immune cells and BMSCs for BTE biomaterials and provided thoughtful insights into the modulation of the inflammatory microenvironment during bone tissue healing, signifying the importance of osteoimmunology in the preparation of bone scaffolds.

## GelMA-based vascularized bone scaffolds

**Fig. 5:**
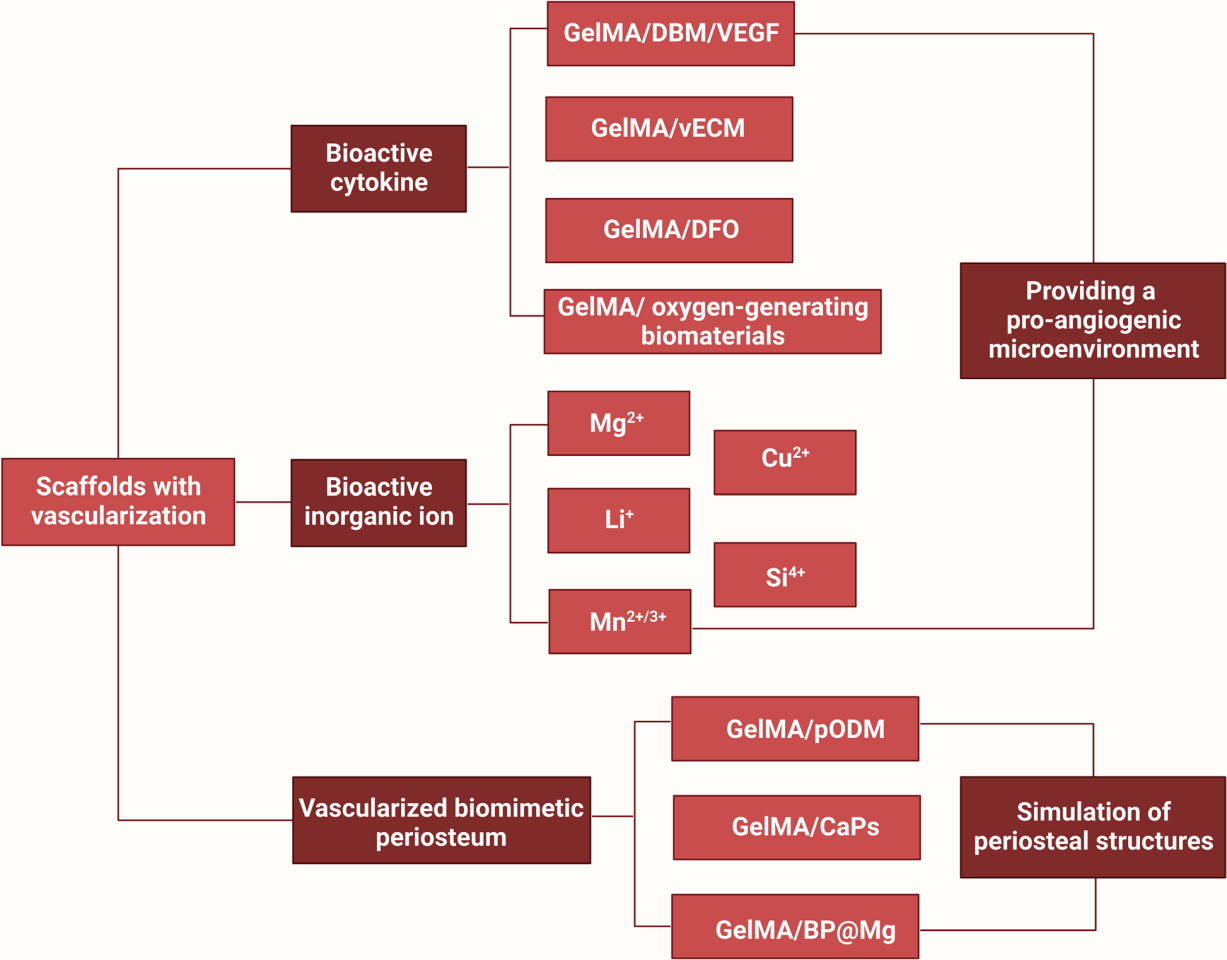
Schematic description of GelMA-based vascularized bone scaffolds (created with BioRender.com)

Natural bone is regarded as a highly vascularized tissue that depends on the condition and distribution of vasculature for necessary blood and nutrients exchange to keep the skeletal integrity and metabolism homeostasis. Graft revascularization is considered a decisive factor affecting the success of bone tissue regeneration, thus, bioactive bone scaffolds with the capability for promoting vascularized bone regeneration are urgently needed for current BTE. GelMA-based hydrogel bone tissue repair materials often require the addition of pro-vascularization and neuroregenerative growth factors to overcome the disadvantage of facile growth factor inactivation, which in turn promotes revascularization, provides abundant oxygenated nutrients for the osteogenesis of newly grafted bone, activates osteoblasts, and accelerates the bone repair process [77]. In this subsection, the incorporation or local transfer of bioinorganic ions in hydrogels remains a popular research topic and, as a strategy, has a heightened capability to promote osteogenesis and angiogenesis. These ions are usually essential cofactors for the synthesis of enzymes, coenzymes or cofactors, actively participate in ion channels, or directly stimulate or mimic secondary signaling processes [78, 79]. Moreover, the application of biomimetic periosteal materials is becoming a popular method for promoting bone tissue vascularization and osteogenesis by mimicking periosteal structures (Fig. 5).

### Bioactive cytokine-incorporated composite hydrogels

Vascularization is an essential part of bone defect healing, which facilitates the nutrient delivery and cell proliferation required for bone remodeling, as well as the regulation of signaling molecules during bone regeneration. In the field of BTE, effective vascularization not only increases scaffold survival, but also effectively promotes the bone regeneration process [80, 81]. To enable rapid and efficient vascularization of bioengineered bone substitutes, introduction of bioactive cytokines to improve the bone tissue microenvironment is a feasible strategy [80]. Herein, we summarized some strategies that act like bioactive cytokines to improve angiogenesis, such as VEGF, decalcified bone matrix (DBM), ECM, DFO, and some bioactive materials for improving hypoxic microenvironments (oxygen-generating materials). By fabricating bioactive cytokine-incorporated composite hydrogel scaffolds with vascular regenerative properties to induce bone tissue regeneration, the shortcoming of insufficient vascularization and osteogenic capability of pure GelMA scaffold was intelligently resolved. 

#### GelMA/HAMA/DBM/VEGF hydrogel

Based on a report by Hao et al. a BTE material consisting of two main components for renovating large bone defects was prepared, and the main component was a DBM scaffold, which is an ideal candidate for bone regeneration due to its natural bone structure, mineralized components, satisfactory cytocompatibility or biocompatibility and osteogenic capacity [28]. Nevertheless, because of its large and irregular internal pore structure, it is not conducive to cellular implantation, and a large amount of its osteoinductive components are lost during the preparation process, resulting in limited application in large bone defect repair [82]. In this study, these disadvantages of the DBM were appropriately addressed, and a hydrogel microsphere consisting of GelMA, HAMA, DBM and VEGF was prepared by microfluidic technology to simulate the osteogenic microenvironment. Bone marrow interstitial stem cells were inoculated on its surface and cultured in vitro to construct bone regeneration units (BRUs), which were incorporated into the DBM scaffold to form a composite decalcified bone framework material for large bone defect repair. The experimental results showed that the BRUs could be injected directly into the subcutis of nude mice, and the BRUs were able to regenerate mature bone tissue after 8 weeks of implantation, including a large amount of bone-specific ECM deposition, clear bone trabecular structures, abundant vascular growth into the bone, and successful ectopic bone regeneration. This strategy not only overcame the shortcomings of injectable BRUs in terms of 3D morphology control and mechanical maintenance but also effectively solved the problems of the low cell inoculation rate and lack of an osteomimetic microenvironment of the DBM scaffold material [28]. This study provided a novel approach to prepare BRUs based on microgels composed of both osteogenic ingredient DBM and VEGF for better biomimic of bone healing microenvironment via photocrosslinable and microfluidic methods, which could be beneficial to overcome the low cell seeding efficiency and poor osteoinductive ability that greatly restrict the application of DBM-based biomaterials in large-sized bone restoration.

#### vECM/GelMA hydrogel

It is still a great challenge to integrate multiple biological processes for satisfactory neovascularization during new bone formation, in the remodeling and recovery of large-sized bone defects in current therapeutic methods in clinics. Considering the pivotal role of vascularized osteogenesis in bone regeneration and the intrinsic angiogenic property of vascular-derived ECM (vECM), Chen et al*.* reported a hybrid vECM/GelMA-based hydrogel delivery platform with a highly simulated 3D structure of natural blood vessels to enhance the therapeutic index of bone morphogenetic protein 2 (BMP-2) and the vascularized microenvironment during bone healing [30]. It has been confirmed that vECM contains numerous of endogenous angiogenic growth factors, and the composition and structure of vECM could regulate endothelial cell adhesion, migration and proliferation, exhibiting strong angiogenic properties [83]. Therefore, the regulation of BMP-2 release kinetics that matched the bone healing time frame and improvement of in vivo angiogenesis were expected to be completed simultaneously. vECM is necessary for BMP-2 delivery systems to meet two key requirements, a high loading efficiency and a sustained release profile, and experimental results showed that those using vECM have a high BMP-2 loading efficiency and slow release. In terms of proangiogenesis, the results of the tube-forming assay showed that the GelMA-vECM group formed more capillary structures, and a significant upregulation of CD31, a representative angiogenic marker, was observed in the GelMA-vECM group compared with the other tested groups. In addition, it also showed good osteogenic ability in osteogenic differentiation-related experiments. In vivo results showed the successful bridging of cranial defects to new bone in the GelMA-vECM@BMP-2 group, with large areas of new bone growth at the periphery of the defects [30], suggesting that this multifunctional hybrid scaffold holds promise for the preparation of regenerative implants with desirable properties to achieve vascularized osteogenesis and bone formation.

#### GelMA-DFO hydrogel

**Fig. 6:**
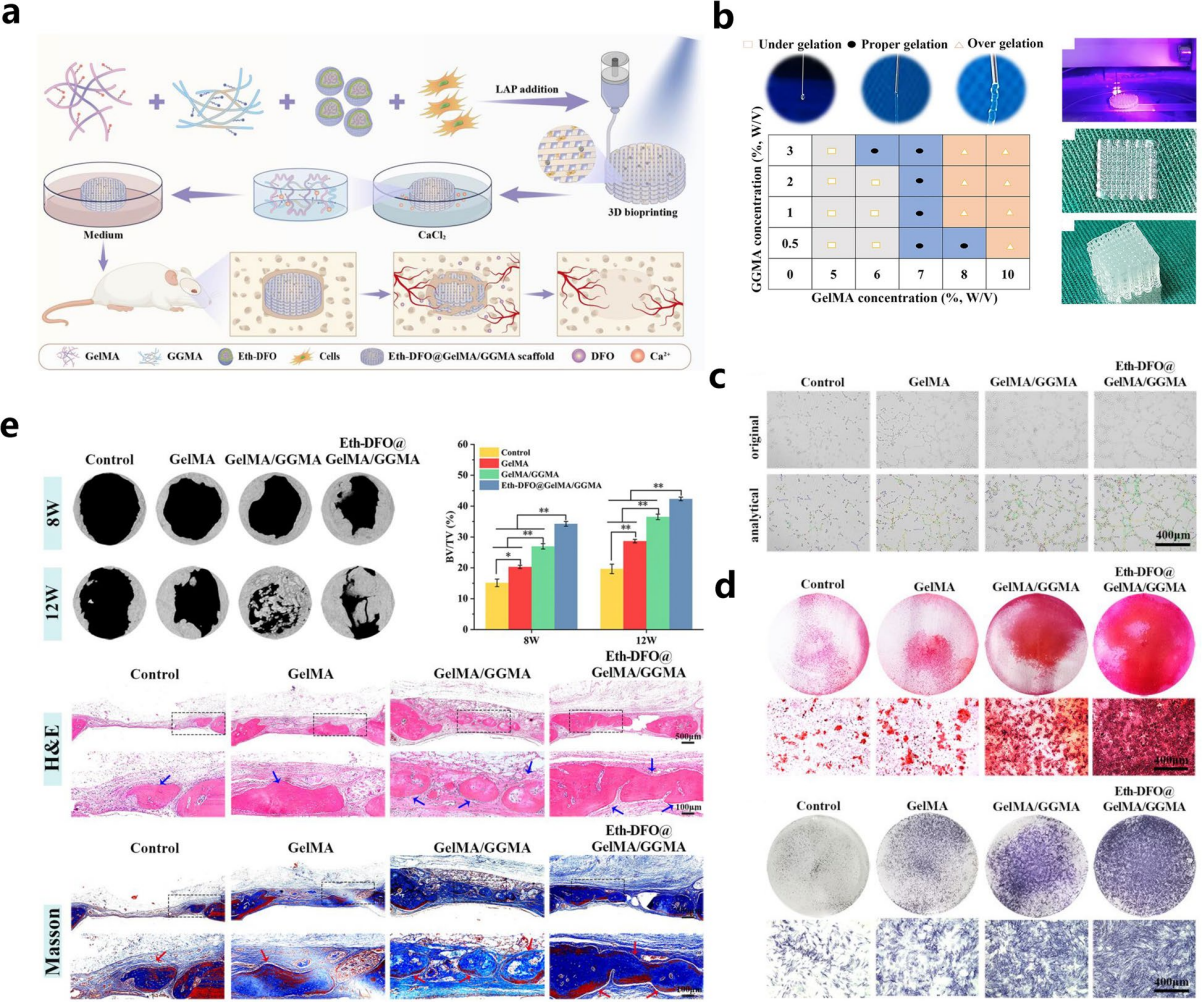
GelMA/CGMA hybrid hydrogels incorporated with DFO/Eth that couple angiogenesis and osteogenesis for vascularized bone regeneration. **a** Graphic description of the 3D bioprinting and experimental procedures of the Eth-DFO @GelMA/GGMA hydrogel scaffold. **b** The suitability and 3D bioprinting process of the hydrogel scaffold. **c** In vitro angiogenic abilities of HUVECs with different hydrogel scaffolds. **d** In vitro osteogenic abilities of different hydrogel scaffolds. **e** Vascularized bone formation induced by the Eth-DFO@GelMA/GGMA hydrogel scaffold in a rat skull defect model at 8 and 12 weeks after surgery. Images reproduced from [29], © 2022 Elsevier Ltd

To improve the shortcomings of early angiogenesis and poor osteogenesis of biomaterial scaffolds in clinical applications, Li et al. combined alcohol-containing liposomal ethosomes (Eth) containing DFO with GelMA/gellan gum methacrylate (GGMA) hybrid bioink to form a 3D-printed scaffold by photocrosslinking and ionic crosslinking, and this bone scaffold promoted angiogenesis and bone regeneration [29] (Fig. 6). In this BTE scaffold, Eth was introduced to achieve slow DFO release with the aid of a protein-polysaccharide bioink and microcarrier-based sustained drug release strategy. Eth are novel liposomes with high deformability and a high guest encapsulation rate that can deliver drugs to the circulatory system more efficiently than ordinary liposomes, followed by the significantly improved intracellular delivery of hydrophilic and lipophilic drugs. In this study, well-maintained DFO bioactivity and local drug concentration and non-observed tissue toxicity under high drug concentrations were recorded after the incorporation of DFO-loaded Eth into the hydrogel scaffolds. The results from the in vitro experiments demonstrated that the Eth-DFO@GelMA/GGMA scaffold exhibited acceptable cytocompatibility with slow DFO release and clearly improved endothelial cell migration and tube formation, mineralized matrix deposition and osteoblast alkaline phosphatase expression. In addition, improved angiogenesis and bone regeneration in a rat cranial defect model were achieved by activating the HIF1-α signaling pathway [29]. In summary, this biodegradable 3D-bioprinted Eth-DFO@GelMA/GGMA scaffold can induce both angiogenesis and osteogenesis to effectively promote bone defect repair, and the combination of GelMA and GGMA hydrogels via an ionic crosslinking reaction produced a double-crosslinked network within the scaffolds with significantly improved mechanical properties. Thus, it is highly recommended to incorporate other types of polymers to prepare GelMA-based bone substitutes to achieve suitable mechanical support and better bioactivities.

#### GelMA/ oxygen-generating biomaterials hydrogels

Bone defects are often accompanied by disruption of the blood supply, resulting in the formation of a hypoxic microenvironment in localized tissues. Subsequently, a hypoxic environment inhibits the ability of BMSCs to differentiate into osteoblasts and promotes the production of proinflammatory mediators such as reactive oxygen species (ROS) [84]. Therefore, oxygen supply and ROS removal from bone defects play an important role in the regeneration of bone tissue. Sun et al. designed a kind of ROS-scavenging, responsive prolonged oxygen generation hydrogel (CPP-L/GelMA) consisting of the antioxidant enzyme catalase (CAT) and ROS-responsive oxygen-releasing nanoparticles (PFC@PLGA/PPs), coloaded liposomes (CCP-L) and GelMA hydrogel [31]. Under hypoxic conditions, CPP-L/GelMA releases CAT-degraded hydrogen peroxide to generate oxygen and continuously releases oxygen for more than 2 weeks triggered by excess ROS. In addition, CPP-L/GelMA promoted the proliferation of human umbilical vascular ECs (HUVECs) in a low oxygen environment, and the prolonged oxygen-enriched microenvironment created by CPP-L/GelMA hydrogel significantly promoted angiogenesis and osteogenesis and inhibited osteoblasts, and favorable bone regeneration in a mouse cranial defect model was recorded after the implantation of the CPP-L/GelMA hybrid hydrogel. In another work reported by Hassan and the colleagues, the effects of calcium peroxide (CPO)-based oxygen-generating microparticles (OMPs) on the osteogenic fate of human BMSCs (hBMSCs) in a severely hypoxic microenvironment were explored and confirmed [32]. Three kinds of hydrogels were constructed: GelMA hydrogels containing osteoinductive silicate nanoparticles (SNP hydrogels), OMPS (OMP hydrogels), or SNP and OMP (SNP/OMP hydrogels). The results showed that the OMP hydrogel was able to promote osteogenic differentiation under hypoxic conditions, and bulk mRNAseq analyses indicated that OMP hydrogels regulate osteogenic differentiation more strongly than SNP/OMP or SNP hydrogels both in normal and hypoxic conditions. Moreover, the subcutaneous implantation assay showed that the OMP hydrogel significantly promoted in vivo angiogenesis. In summary, the addition of OMPs improved the hypoxic environment and promoted early neovascularization and later osteogenic differentiation, which has a great promise for bone tissue engineering. These studies as discussed above offer alternative intelligent responsive oxygen-releasing bone tissue scaffolds by incorporating oxygen-generating bioactive materials for effective bone regenerative applications required prolonged oxygen supply under oxygen-deprived circumstances, which demonstrated great clinical therapeutic potential in large bone defects resulting from severe bone fractures, chronic inflammation or diabetes mellitus (DM)-related bone destruction.

### Bioactive inorganic ion-incorporated composite hydrogels

It is still a great challenge to obtain large vascularized bone tissues during the healing of bone defects, and incorporating bioactive inorganic ions with osteogenic and angiogenic functions into materials is currently a type of practicable strategy in vascularized bone tissue engineering [80]. To sum up, a number of ionic components have an affirmative impact on the vascularization and bone regeneration, allowing for the accurate ions delivery to functionalize the bone scaffolds for vascularized bone healing. Some inorganic ions such as manganese ions (Mn^2+/3+^), act as essential cofactors of enzymes that can activate ion channels or secondary signaling, influencing vascularization and bone regeneration [79]. Other inorganic ions, such as magnesium ions (Mg^2+^), lithium ions (Li^+^), silicon ions (Si^4+^) and copper ions (Cu^2+^), have also been reported to promote angiogenesis and osteogenesis [85]. The following are several representative scaffolds incorporating bioactive inorganic ions as previously mentioned.

#### GelMA-BP-Mg hydrogel

**Fig. 7:**
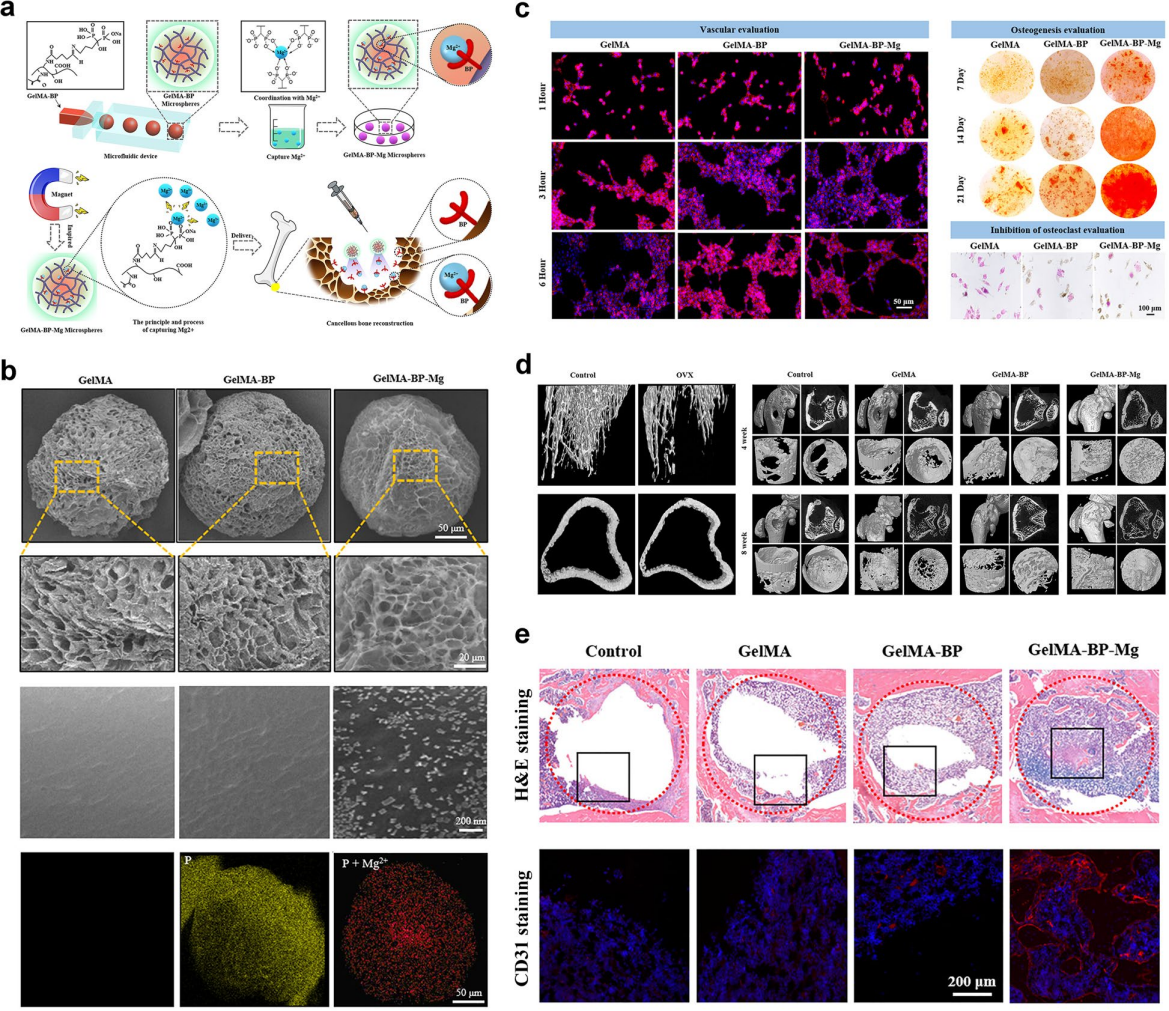
BP-modified injectable hydrogel microsphere system (GelMA/BP/Mg) with strong Mg^2+^-trapping ability for the treatment of osteoporotic bone defects. **a** Graphical illustration of the construction of GelMA-BP-Mg microspheres and capturing principle of Mg^2+^. **b** Surface characterizations of the prepared microspheres as confirmed by SEM. **c** In vitro vascularization, mineralization, and osteoclast inhibition of the composite microspheres. **d** Micro-CT-based radiographic analysis of the promoted cancellous bone regeneration induced by the injectable hydrogel microspheres (GelMA-BP-Mg) in rats with osteoporotic bone defects at 4 and 8 weeks after surgery. **e** Histological observation of the in vivo new bone formation and vascularization of different composite microspheres. Images reproduced from [33], © 2021 American Chemical Society

Magnesium ions (Mg^2+^) play a critical role in bone growth and development, promoting bone healing mainly by activating osteoblast differentiation, restricting osteoclast functions, and strengthening the adhesion of human bone-derived cells. Moreover, Mg^2+^ effectively facilitates of angiogenesis both in vitro and in vivo [86]. Consequently, Mg^2+^ serves as a favorable bone-forming factor and is widely used in bone tissue repair engineering. Recently, most magnesium-based bone repair materials have been combined with magnesium metal by passive methods, which unavoidably lead to quick and uncontrollable magnesium release; thus, a "high-magnesium microenvironment" can easily form around the implant, which causes adverse inflammation or even toxic damage to the physiological activities of the surrounding normal cells and tissues [87]. To enhance the sustained release of Mg^2+^ by composite materials, Zhao et al. fabricated a bisphosphonate (BP)-modified injectable hydrogel microsphere (GelMA-BP-Mg) system by grafting BP onto GelMA microspheres, which resulted in strong Mg^2+^-trapping ability and slow BP release through coordination reactions [33] (Fig. 7). BP has bone-targeting properties, and its slow release enhances its ability to activate osteoblasts and ECs and inhibit osteoclasts, ultimately leading to cancellous bone reconstruction. The sustained release of BP and Mg ions from the composite microspheres was observed. Regarding the angiogenic properties of the composite microspheres, this synthesized GelMA-BP-Mg hybrid scaffold significantly promoted in vitro tube formation and angiogenesis-associated gene expression (endothelial nitric oxide synthase (e-NOS) and VEGF). The GelMA-BP-Mg group showed better osteogenesis in vitro. In addition, regarding the bone-targeting ability assay of BP, the osteoclast inhibition assay showed that the number of tartrate-resistant acid phosphatase (TRAP)-positive multinucleated osteoclasts was significantly reduced in the GelMA-BP-Mg, indicating the inhibitory effect of the hybrid scaffold on osteoclastogenesis. In vivo findings from a rat model of osteoporosis demonstrated that the GelMA-BP-Mg group significantly promoted cancellous bone reconstruction with the effective capture of Mg^2+^ in the body. Considering the multiple functions of GelMA-BP-Mg hybrid hydrogels concerning osteoblastogenesis, osteoclastogenesis and angiogenesis, this study presents an effective approach for treating osteoporotic bone defects in patients with osteoporosis by capturing Mg^2+^.

#### GelMA/Li-MBG hydrogel

Mesoporous BG nanoparticles (MBNs) are often used as drug delivery vehicles or bioinjection materials due to their unique processing properties, large specific surface area, high drug loading capacity and good release properties. It is possible to obtain MBNs with different properties that possess different biological effects by regulating the corresponding ratio and adding elements [88, 89]. However, MBN-loaded bone repair materials are rigid and do not contain bone immunomodulatory factors, making them incapable of regulating chronic inflammation in the diabetic microenvironment and coordinating bone regeneration [34]. It has been reported that lithium ions (Li^+^) facilitate BMSC proliferation and promote osteogenesis by regulating autophagy, and other studies have reported the role of Li^+^ in macrophage polarization, where it can effectively inhibit the inflammatory response [90, 91]. A Li^+^-functionalized BG hydrogel scaffold for combating diabetic bone regeneration exhibited sustained Li^+^ release in the diabetic microenvironment and evidently promoted bone formation. Experimental results revealed that the hydrogel was mechanically adaptive to complex defect shapes. These Li^+^-modified BG hydrogels could promote cell proliferation, direct osteogenesis and modulate macrophages in a high-glucose (HG) microenvironment in vitro and indirectly stimulate osteogenesis and neovascularization through BMP-2 and VEGF secretion. Furthermore, the GelMA/Li-MBG scaffold released Li^+^ to alleviate inflammation in the femoral defects of diabetic rats, leading to an anti-inflammatory microenvironment for in vivo osteogenesis and angiogenesis and thus promoting diabetic bone regeneration [34]. Although great efforts have been made to clarify the complicated interactions among immunomodulation, bone regeneration and vascularization in the diabetic microenvironment, the treatment of diabetic bone defects remains challenging and unsatisfactory. Based on the excellent performance of Li^+^ in anti-inflammation, osteogenesis and angiogenesis, this Li^+^-incorporated BG/GelMA hydrogel scaffold with favorable immunomodulatory properties could be a promising candidate for accelerating diabetic bone healing in future clinical practice.

#### GelMA-PEGDA/SiPAC hydrogel

It has been found that silica released from bioengineered bone substitutes significantly improved angiogenesis and accelerated bone regeneration [92], and phosphorus, an important bone component, plays a pivotal role in the bone repair process, inducing mineralization and promoting bone regeneration [93]. In addition, several 2D nanomaterials, such as MoS_2_, MXene and BP nanosheets, have been reported to provide multifunctional cues for BTE-based bone repair strategies. Xu et al. explored the angiogenic and osteogenic roles of a novel bioactive and biodegradable nanomaterial, 2D silica-phosphorus (SiP), and found that the incorporation of SiP nanosheets into biocompatible hydrogels did not undermine the mechanical performance of the composite materials [35]. In this study, acryloyl chloride (AC) was used to modify SiP nanosheets with vinyl-linked surfaces. The results showed that GelMA-polyethylene glycol diacrylate (PEGDA)/SiPAC biohybrid hydrogels had good biocompatibility and biodegradability and sustained the slow release of Si and P ions. In addition, the GelMA-PEGDA/SiPAC hydrogels further enhanced the osteogenesis of MSCs as well as HUVECs. The released bioactive ions mentioned above acted as angiogenic stimulators to activate VEGF, bFGF and CD31 expression to promote angiogenesis and induce BMP-2 and OCN expression to facilitate the osteogenic differentiation of MSCs and promote bone regeneration. Furthermore, the biohybrid hydrogel scaffold doped with SiP nanosheets showed excellent osteogenic induction and revascularization ability in an in vivo rat cranial bone defect model, confirming the function of the multifunctional SiP nanocomposite scaffold as a mediator of osteogenesis and angiogenesis in one system. Based on these findings, GelMA-PEGDA/SiPAC hydrogel scaffolds with typical 3D interconnected structures and attractive bioactivities demonstrated great potential in vascularized bone reconstruction. This study was the first report that attempted to explore 2D SiP as a bioactive and biodegradable nanomaterials for vascularized bone regeneration, and it provides a multifunctional SiP nanocomposite hydrogel scaffold with regulatory effects on osteogenesis and angiogenesis in one system, signifying the bright prospect of 2D nanomaterials in tissue engineering and regenerative medicine.

#### GelMA/other bioactive inorganic ions hydrogels

**Fig. 8:**
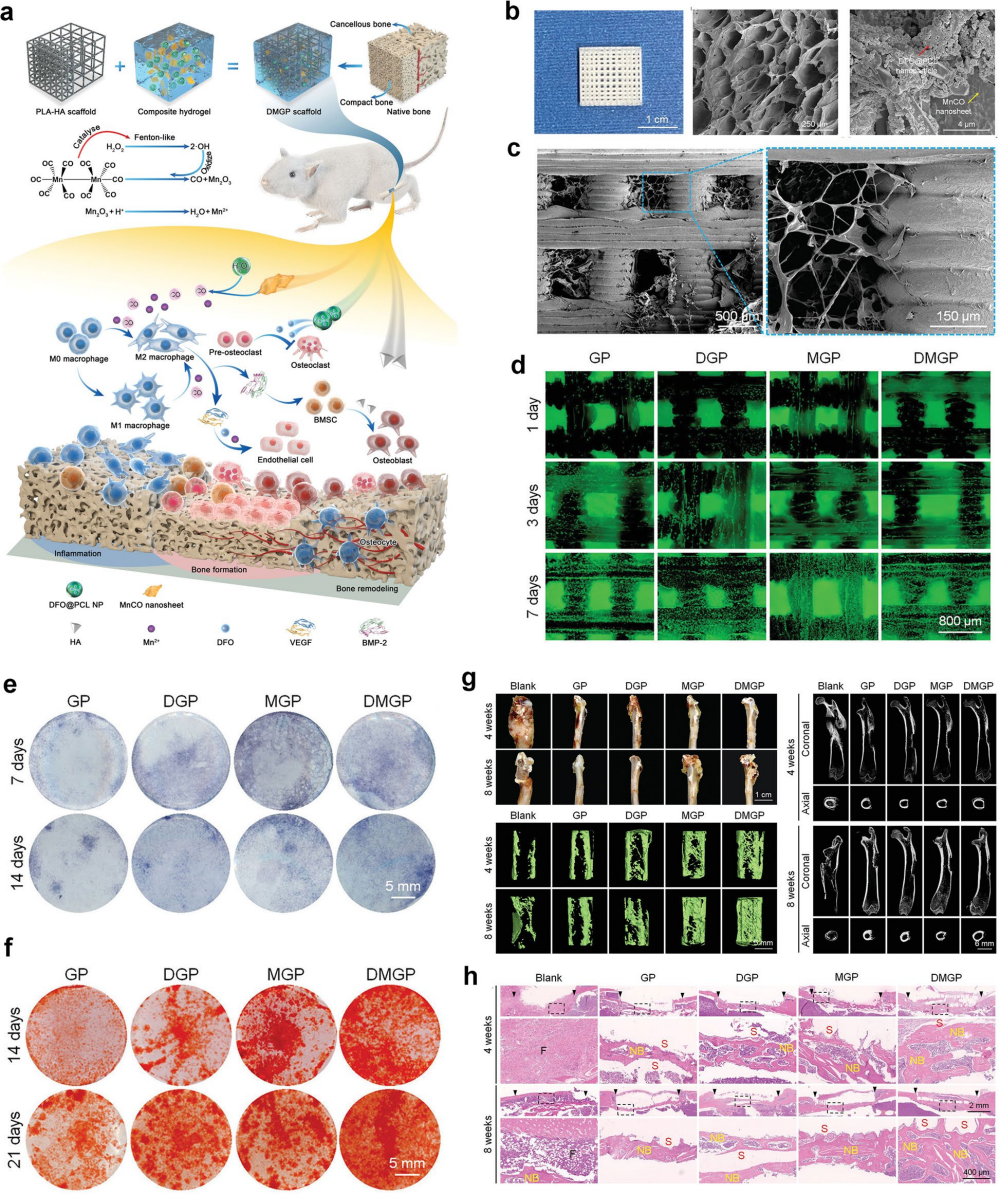
Osteoimmunity-regulating hierarchical hybrid scaffold with excellent immunomodulatory, angiogenic and osteogenic properties for large-scale bone defect repair. **a** Schematic illustration of the preparation of PLA/HA framework integrated with DFO and MnCO nanomaterials in GelMA hydrogels and its osteoimmunomodulatory effects. **b** Surface characterization of PLA/HA scaffold sand GelMA/DFO/MnC composite hydrogel. **c** SEM images of DMGP hierarchical hybrid scaffold. **d** In vitro cytocompatibility of DMGP scaffold towards BMSCs via Live/Dead staining. **e**, **f** In vitro osteogenic activity of BMSCs on DMGP scaffold via ALP and ARS staining. **g** In vivo bone regenerative potential of DMGP composite scaffold in a critical-sized femoral bone defect model as confirmed by microCT reconstruction. **h** Histological evaluation (H&E staining) of new bone formation induced by DMGP scaffold at 4 and 8 weeks after surgery. Images reproduced from [36], © 2022 Wiley–VCH GmbH

In addition to the magnesium and lithium ions as mentioned above, there are also some other bioinorganic ions that can improve the osteogenic microenvironment and promote angiogenesis, such as manganese and copper ions. Manganese ions (Mn^2+/3+^) play an important role in osteogenesis and angiogenesis, and one of the important players involved in these biological functions is manganese superoxide dismutase (MnSOD), which can protect the mitochondrial components of cells from reactive oxygen species damage [85]. Taking advantage of the unique property of manganese ions, Zhang and the colleagues prepared a 3D printed hybrid hydrogel scaffold composed of DFO and MnCO nanosheets with poly lactic acid (PLA)/HA framework as biomimetic natural bone ECM (DMGP), which served as an osteoimmunity-regulating BTE scaffold for effective bone regeneration due to evident immunomodulatory, angiogenic and osteogenic capabilities [36, 94] (Fig. 8). In vitro experiments showed that manganese ions and DFO blocked the degradation of HIF-1α and activated the HIF-1α pathway, initiated a hypoxic microenvironment and further promoted neovascularization, together with an inhibitory effect on osteoclast differentiation, indicating favorable osteoimmunomodulatory properties for accelerating sizeable segmental bone defects in future clinic scenarios. This study emphasized the significant role of osteoimmunomodulation as a novel therapeutic strategy for facilitating osteoimmune balance between immune or bone cells and implanted biomaterials. Similarly, copper ions are a class of bioinorganic ions that promote angiogenesis, which is induced mainly through the VEGF and HIF-α pathways [85, 95]. Several studies have utilized the pro-angiogenic effect of copper ions to apply them to the repair of bone defects. Xu et al*.* designed a GelMA hybridized hydrogel to promote vascularized bone regeneration which containing copper ion-modified germanium-phosphorus (GeP) nanosheets that served as neuro-vascular regeneration and antimicrobial agents. As expected, such a newly designed biohybrid hydrogel scaffold continuously and slowly releases copper ions, promoting osteogenic differentiation and angiogenesis, leading to promoted bone regeneration in a rat cranial defect model. In addition, this innovative GelMA/GeP@Cu hybrid hydrogel demonstrated promising potential in improving neuro-vascularized bone regeneration and eliminating bacterial infections [37]. Based on these findings, the incorporation of bioinorganic ions, such as copper or manganese ions, further enhances the osteogenic and vasculogenic capabilities of GelMA hydrogels, which are an integral part of BTE.

### GelMA-based composite hydrogels for vascularized biomimetic periosteum

The periosteum plays an important role in bone regeneration due to its rich blood supply and osteogenic activity. As a significant source of local growth factors and capillary system that recruit osteogenic cells, periosteum facilitates the osteogenesis under the action of mechanical stimulation via the regulation of Wnt and BMP signaling pathways [96]. In recent BTE, tissue-engineered periosteum has been increasingly investigated to promote the repair of bone defects through biomimetic technology, which makes its function and structure similar to those of natural periosteum [96]. More importantly, periosteum-induced osteogenesis and vascularization are more close to the actual healing process of bone repair, and tissue-engineered periosteum deserves in-depth investigation prior to clinical practices, and the application of periosteum structure and components within the bioengineered scaffolds is also increasingly diversified and versatile to meet different types of bone repair. In this context, we present some bionic periosteal scaffolds based on GelMA hydrogel below, and the material composition and bioactivities were summarized and discussed.

#### pODM/GelMA hydrogel

As one of the critical components of bone tissues, the periosteum not only provides indispensable blood supply to the bone cortex but also provides the skeletal progenitor cells involved in bone formation; therefore, periosteal integrity is critical for fracture healing and bone regeneration [97]. Bone defect repair by mimicking the structure or function of the periosteum is also a current research direction in BTE. The ECM is a network of proteins and proteoglycans as well as growth factors and other signaling molecules that has great potential as an artificial bone membrane. Decellularized ECM derived from animal tissues has been widely utilized as an osteoconductive matrix for bone repair, but its development has been limited by several drawbacks, such as inconsistent composition and structure due to the diversity of sources and possible immune rejection by host tissues [98, 99]. Therefore, decellularized ECM obtained from cultured cells is beginning to attract interest for application in tissue regeneration. Yu et al. constructed an engineered periosteal-bone substitute by using preosteoblast-derived matrix (pODM) as a mimetic bone membrane wrapped in a GelMA hydrogel [38]. The cell-free pODM sheet used was obtained by culturing MC3T3-E1 cell sheets on poly(dimethylsiloxane), followed by decellularization for subsequent in vitro osteogenic differentiation and in vivo bone reconstruction. The results showed that BMSCs on the pODM sheet exhibited good adhesion and proliferation on pODM. In addition, the pODM sheets showed concentration-dependent chemotaxis toward BMSCs and promoted the osteogenic differentiation of BMSCs. In the repair of large bone defects, the constructed engineered periosteal-bone substitute (pODM/GelMA) showed good bone repair effects on segmental rabbit radius bone defects. The critical-sized bone defects were completely healed, and the medullary cavity of the radial diaphysis was significantly and completely reconstructed after 12 weeks of pODM/GelMA hydrogel implantation, suggesting the importance of combining pODM with GelMA as a biomimetic bone substitute for bone repair.

#### CaPs@GelMA-F hydrogel

To fabricate artificial bone membranes with the ability to accelerate angiogenesis and osteogenesis in defect regions, Liu et al. used the electrostatic spinning technique to prepare an organic‒inorganic hybrid bionic bone membrane that could induce in situ mineralization and control long-term ion release in localized regions [39]. The fibrous scaffolds prepared by the electrostatic spinning technique exhibited a high surface-to-volume ratio and adjustable pore size, properties that allowed them to effectively mimic the structure of the capillary system in the periosteum. In addition, the morphology of these nanofibers was similar to that of collagen fibers in bone, leading to effective interactions with stem cells and stimulation of autocrine/paracrine growth factor signaling pathways, thereby promoting bone regeneration. Calcium ions and phosphate released from calcium phosphate nanoparticles (CaPs) are important inorganic ions in the bone formation process, but CaPs cannot stay localized for a long time due to their nanoscale diameter and the flow of body fluids, whereas combining them with electrostatic spinning technology can solve this problem effectively. CaPs were prepared by the emulsion method and then further doped with GelMA by electrostatic spinning fibers to construct hybridized hydrogel fibers (CaPs@GelMA-Fs). The authors observed the sustained release of calcium ions lasting for 14 days, and the CaPs@GelMA-F surface showed significant mineralization. Then, the biocompatibility of the hybridized fibers was shown by coculture with MC3T3-E1 cells. Finally, by coculture with HUVECs and MC3T3-E1 cells, the CaPs@GelMA-Fs exhibited the potential ability to promote angiogenesis and osteogenesis, making it possible to accelerate bone repair by the application of this biomimetic organic–inorganic hybrid hydrogel electrospun periosteum.

#### GelMA-BP@Mg/GelMA-PEG-β-TCP hydrogel

**Fig. 9:**
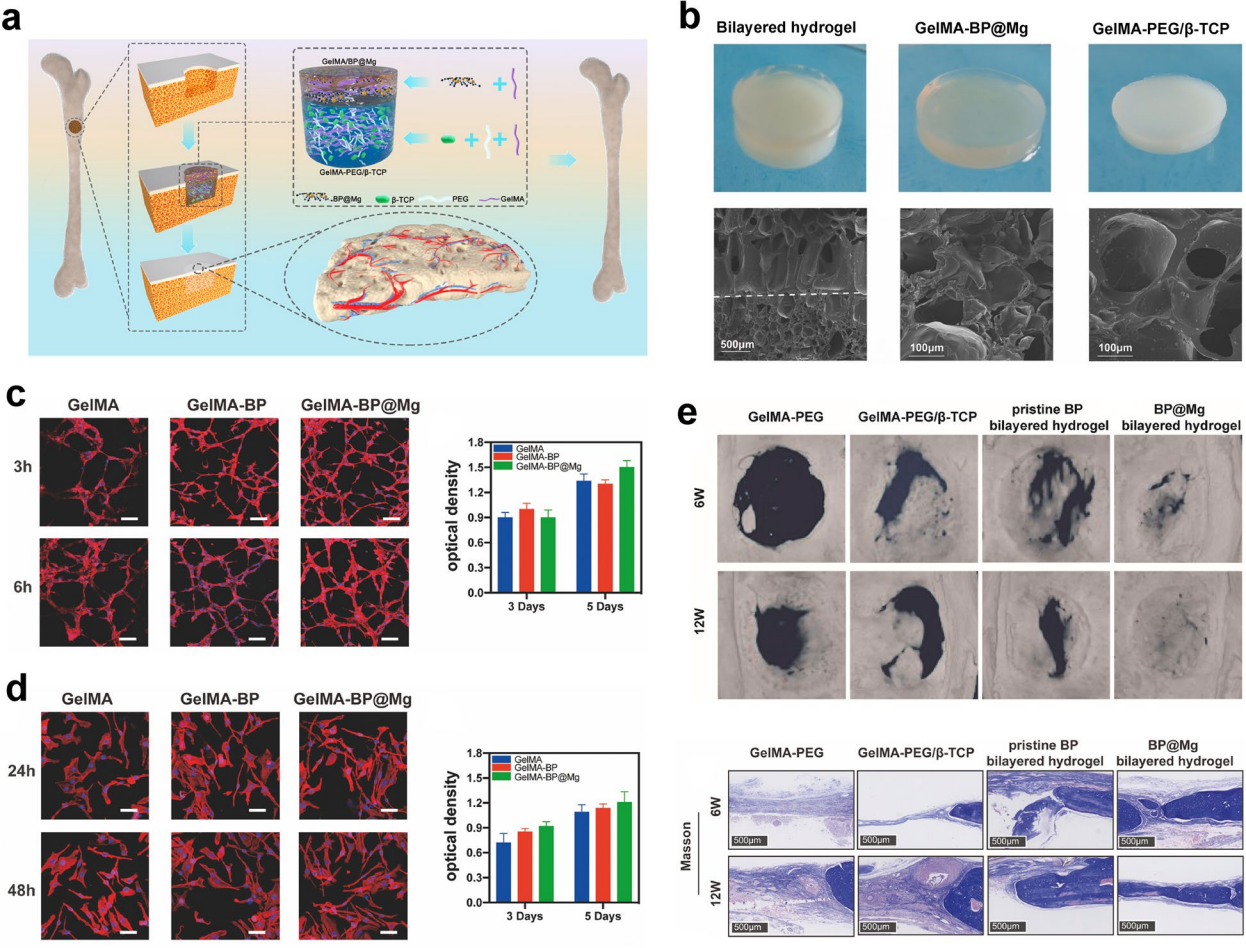
Stratified-structural hydrogel scaffold integrated with Mg ions-loaded BP nanosheets with good angiogenesis, osteogenesis and upregulation of nerve-related protein expression for promoting neuro-vascularized bone regeneration. **a** Schematic illustration of the bilayer hydrogel scaffold consisting of the upper (GelMA-BP@Mg) hydrogel and the bottom layer (GelMA-PEG/β-TCP) hydrogel. **b** Characterization of the bilayer hydrogel scaffolds. **c** In vitro angiogenesis evaluation of HUVECs on different hydrogel scaffolds. **d** Cell viability of HUVECs cultured on different hydrogels. **e** In vivo bone regeneration of rat calvarial defects treated with different hydrogel scaffolds. Images reproduced from [40], © 2022 Elsevier Ltd

Despite the extensive application of bone substitutes that mimic native bone structures in bone restoration, neurovascularization is usually neglected in the design of BTE scaffolds. Inspired by the significant and irreplaceable role of neurogenesis during the healing process of bone defects, Xu and colleagues designed a bilayer hydrogel platform mimicking bone membranes, with magnesium ion-modified black phosphorus (BP@Mg) nanosheets compounded with GelMA hydrogel (GelMA/BP@Mg) as the top layer and a double-network hydrogel system consisting of GelMA, PEGDA, and β-TCP nanocrystals (GelMA-PEG/β-TCP) as the bottom layer [40] (Fig. 9). BP nanomaterials are a new member of the 2D material family with a direct band gap, excellent electrical conductivity and good biodegradability. The BP degradation product is the phosphate anion, which is a component of bone tissue that facilitates mineralization and accelerates bone repair [93]. Modification with magnesium ions can improve the stability of BP; in addition, magnesium ions can induce angiogenesis and peripheral nerve repair [100, 101]. Therefore, the upper GelMA/BP@Mg hydrogel can be used as a mimetic bone membrane structure to promote angiogenesis and neurogenesis, while the underlying GelMA-PEG/β-TCP hydrogel can promote osteogenic differentiation. The results showed that the expression of angiogenesis-related genes (VEGF, vWF and CD31) and neural stem cell (NSC)-related proteins was significantly increased, which significantly promoted angiogenesis; the underlying hydrogel GelMA-PEG/β-TCP, on the other hand, promoted BMSC activity and osteogenic differentiation. In an in vivo rat cranial defect model, the bilayer hydrogel scaffold group showed good pro-osteogenic properties, and the cranial defect was completely covered by new bone 12 weeks after implantation, providing a design strategy for engineering nerve-vascular network scaffolds to repair bone defects [40]. Considering the unique effects of the peripheral nervous system (PNS) and the secreted neuropeptides (such as substance P and calcitonin gene-related peptide) on osteogenic differentiation, osteoactivity and bone remodeling, it is very important to give more attention to the neurovascularization properties of implanted biomaterials used to support highly effective bone healing.

## GelMA-based bone scaffolds with osteoinductivity

**Fig. 10:**
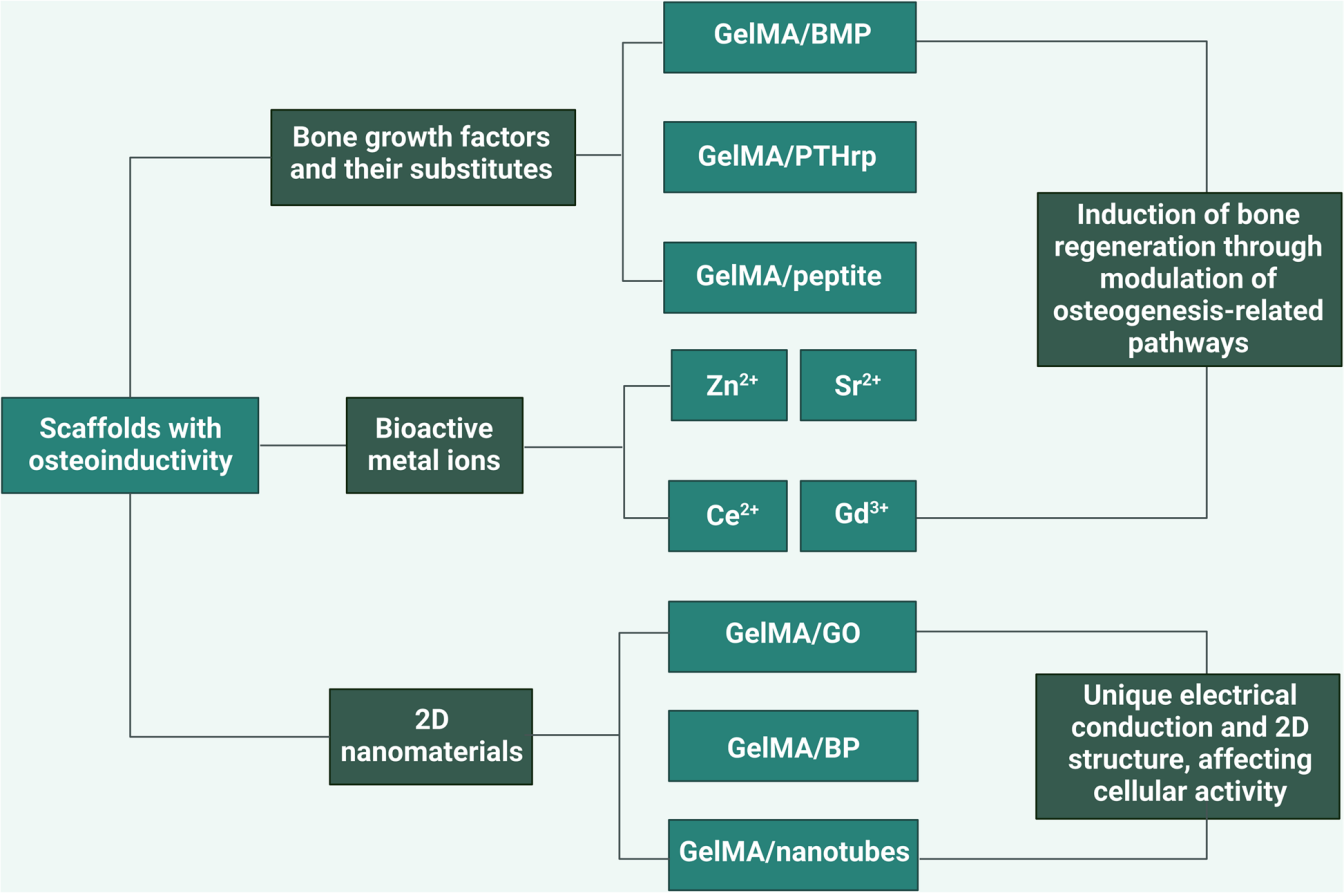
Schematic description of GelMA-based bone scaffolds with osteoinductivity (created with BioRender.com)

The healing process of bone injury involves the regulation of multiple growth factors with pivotal roles in regulating various biological processes, such as bone growth, differentiation, and remodeling [102]. The successful osteoconduction and osteoinduction of implanted bone substitutes are highly dependent on various biological factors. Therefore, the direct application of growth factors has been widely studied and clinically accepted as an effective and feasible therapeutic strategy. In addition, some 2D nanomaterials, such as graphene oxide (GO), BP nanosheets, and nanotubes (NTs), provide a good matrix environment and promote controlled growth factor delivery at different stages of bone regeneration due to their unique structures and physicochemical properties [93, 103, 104]. With the rapid development of BTE, numerous metal ion-modified composite scaffolds have exhibited great application potential in bone defect therapy due to the particular functions of metal ions in osteoinductivity, angiogenesis and anti-infection [105]. GelMA-based bone tissue composite scaffolds loaded with induced bone growth factors, bioactive metal ions or 2D nanomaterials are effective in reducing the healing period of bone defects during the bone repair process (Fig. 10).

### Composite hydrogels with incorporated bone growth factors and their substitutes

In the process of bone healing, the role of osteoinductive factors should not be ignored; they promote the process of bone formation and resorption by regulating related signaling pathways. The main relevant growth factors with osteoinductive capacity are bone morphogenetic proteins (BMPs), parathyroid hormone (PTH)/PTH-related peptide (PTHrP), and their substitutes [106]. BMP increases the expression of Runx-2 and Osx by mediating the Smad and MAPK pathways, promoting osteogenic differentiation of bone marrow mesenchymal stem cells [107]. PTH regulates bone mass in an endocrine manner and has a role in regulating both bone formation and resorption [106]. Therefore, the incorporation of such bone growth factors into GelMA scaffolds can increase the osteoinductive capacity of the scaffolds and accelerate the repair of bone defects. In addition, the long-term instability and possible inactivation of loaded growth factors within the composite hydrogels should also be paid extensive attention to further improve the therapeutic outcomes of these types of hybrid hydrogel bone scaffolds.

#### GelMA/BMP hydrogels

BMPs, especially BMP-2 and BMP-4, are typical members of the transforming growth factor (TGF)-β superfamily that have excellent osteoinductive properties and multiple functions in skeletogenesis, such as mesenchymal condensation, bone morphogenesis, growth plate development, and osteoblast differentiation, and are extensively applied in studies related to bone defect regeneration [107]. Sun et al. reported modified bioinks containing gelatin, GelMA and 4-arm-poly(ethylene glycol) acrylate (PEG) to prepare 3D-bioprinted scaffolds containing BMSCs, RAW264.7 macrophages, and mesoporous silica nanoparticles (MSNs) loaded with BMP-4 [41]. These 3D-bioprinted multicell-laden scaffolds were designated to accelerate diabetic bone defect healing, and MSNs can sustainably release BMP-4, which promotes the polarization of RAW264.7 cells into M2 macrophages, thereby reducing the levels of proinflammatory factors [108]. The BMP-4 released by MSNs and BMP-2 secreted by M2 macrophages together promoted the osteogenic differentiation of BMSCs in these porous scaffolds. In addition, MSNs have a high BMP-4 loading rate and sustained release of BMP-4. The GelMA/gelatin/PEG/MSN scaffolds also demonstrated good biocompatibility, and regarding the polarization of macrophages, the RAW/BMP-4 group showed an increase in M2 cells and a decrease in M1 cells compared to the RAW group. The expression level of BMP-2 and the mineralization degree recorded in the RWA/BMP-4 group and BMSC/BMP-4/RAW group, were significantly greater than those in the other groups during the in vitro osteogenic differentiation assays of BMSCs. In an in vivo diabetic rat cranial defect assay, obvious M2 macrophage polarization and alleviation of the inflammatory microenvironment were observed in defect sites implanted with 3D substitutes containing MSNs/BMP-4, demonstrating significantly accelerated bone repair [41]. Chai et al. reported photocrosslinked composite bioactive scaffolds containing GelMA, BMSCs and BMP2, and this bone substitute exhibited appropriate ability to promote stem cell attachment and proliferation, as well as good biocompatibility and the ability to stimulate the osteogenic differentiation of BMSCs in vitro. In addition, this composite bioactive scaffold with superior osteogenic potency demonstrated higher osteogenic potential than scaffolds equipped with BMSCs or BMP-2 alone in a rat distal femoral bone defect model and could be a candidate material for the clinical repair of irregular bone defects [109].

#### *GelMA/PTHrp (*Abaloparatide*) hydrogel*

PTH is a naturally generated hormone containing 84 amino acids that acts as a modulator of calcium and phosphate homeostasis in the body, and it has been confirmed to promote bone mass and bone strength, thus reducing bone loss. Structure‒function analysis of PTH indicates that these activities largely rely on the N-terminal fragment (including amino acids 1–34, referred to as PTH(1–34)) [110]. Currently, there are two derivatives of PTH: the full-length protein PTH(1–84), commercially known as Natpara, and the partial protein PTH(1–34), known as teriparatide [111]. Abaloparatide is an analog of human PTHrp(1–34) (teriparatide) that shares the same sequence of the first 20 amino acids. Ning et al. used the porous structure of a GelMA hydrogel to construct a drug delivery system for abaloparatide, and the experimental results showed that abaloparatide had an advantage over teriparatide regarding the treatment of bone defects and promotion of bone regeneration [42]. These results indicated that the 3D porous structure of the GelMA hydrogel could effectively prolong the release of abaloparatide (more than 10 days). In addition, in vitro experiments showed that abaloparatide treatment significantly promoted the viability, differentiation and mineralization of preosteoblastic MC3T3-E1 cells. In vivo bone defect experiments showed that the abaloparatide-loaded hydrogel group had better bone regeneration than the blank control group and GelMA group, indicating that GelMA-based hydrogel injection can serve as an alternative treatment for bone defects.

#### GelMA/peptide hydrogels

**Fig. 11:**
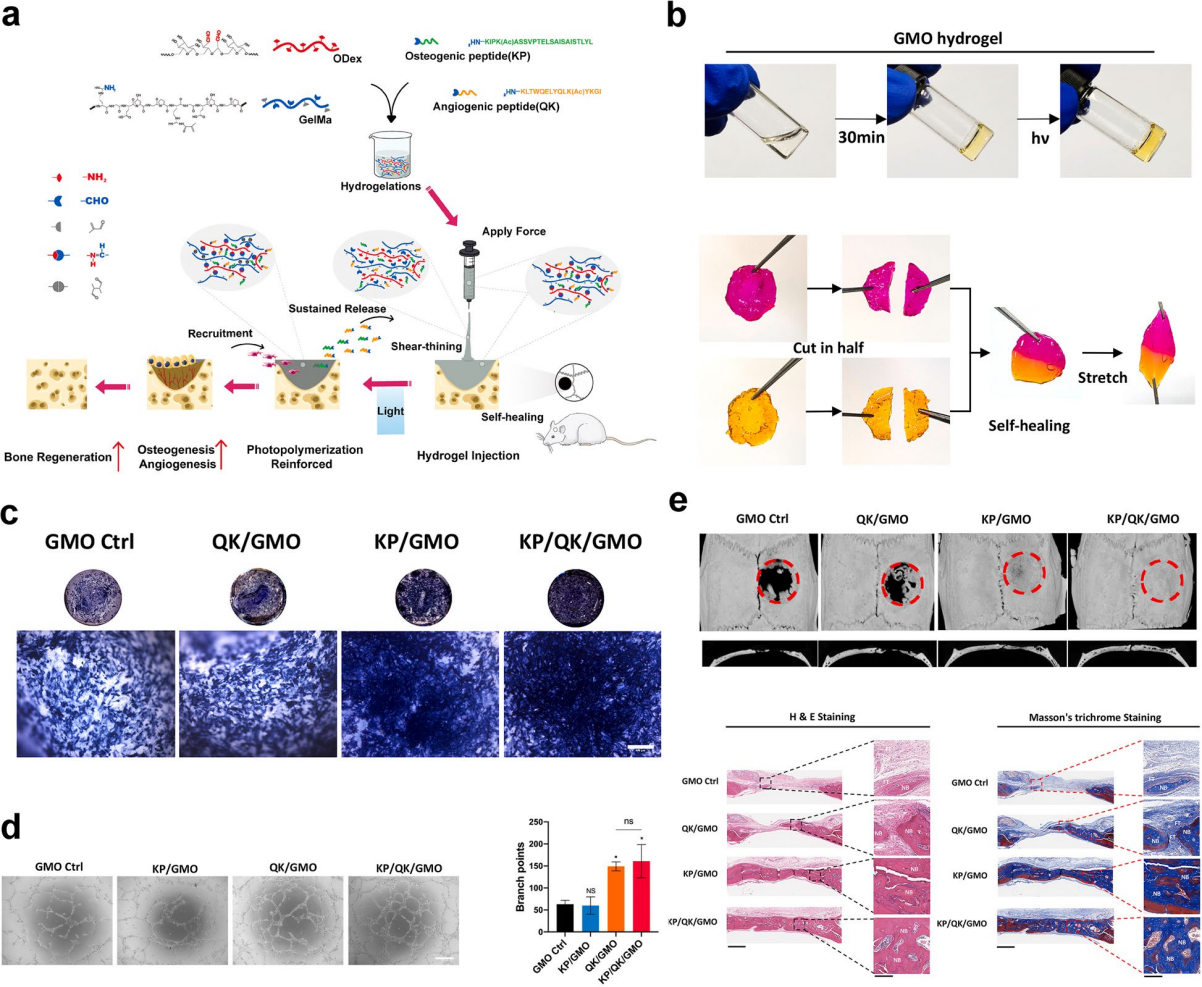
A novel injectable self-healing hydrogel combining synergistic osteogenic and angiogenic effects of KP and QK peptides with GelMA to realize good defect fitting and bone regeneration. **a** Schematic illustration of the synthesis and experimental procedures of the KP/QK/GMO hydrogel scaffold. **b** Optical images of the GMO hydrogel and the self-healing process of the SN-GMO hydrogel. **c** In vitro osteogenic ability of BMSCs cultured on different GMO hydrogels confirmed by ALP staining after 7 days of induction. **d** Tube formation of HUVECs cultured on different GMO hydrogels. **e** In vivo bone regeneration of rat skull defects treated with the KP/QK/GMO hydrogel scaffold. Images reproduced from [44], © 2022 Elsevier Ltd

The incorporation of growth factors into BTE substitutes has been severely limited due to certain drawbacks in their practical application, such as high production costs, immunogenicity and transient effects caused by their short half-life and structural instability [112]. To overcome the easy-inactivation and burst release of polypeptides involved in the in vivo application, Qiao et al. prepared a novel osteogenic peptide hydrogel (GelMA-c-OGP) by crosslinking template gelatin (GelMA) with a photocrosslinkable osteogenic growth peptide (OGP) under UV radiation [43]. The C-terminal pentapeptide (Tyr-Gly-Phe-Gly-Gly) of OGP is the physiologically active form of OGP cleaved by protein hydrolysis, and OGP enhances the proliferation and differentiation of osteoblasts in vitro. In addition, unlike growth factors, OGP has a stable active structure that resists high temperatures and organic solvents. The hydrogel made by co-crosslinking GelMA with c-OGP prevented the abrupt release of OGP and had good mechanical properties, and the experimental findings confirmed the promotion of bone regeneration by this hydrogel delivery system. It is still a challenge in clinical practice to fill irregular bone defects resulting from trauma or surgery with traditional bone substitutes; therefore, Li et al. prepared an injectable and self-healing hydrogel by combining KP and QK peptides with GelMA to realize good defect fitting and minimal invasiveness [44] (Fig. 11). The KP peptide, an osteogenic peptide designed from BMP2, corresponds to the BMP2 joint epitope sequence, and its osteogenic activity has been confirmed through studies. Moreover, the QK peptide, an angiogenic peptide designed from VEGF, corresponds to the VEGF helix sequence and has been used to regulate angiogenesis. This study combined GelMA with oxidized dextran through the formation of dynamic imine bonds to form a GMO composite hydrogel, where the dynamic imine bonds endowed the composite hydrogel with self-healing and shear-thinning capabilities, making it self-healing and injectable. The osteogenic peptide KP and the angiogenic peptide QK were linked to the GMO hydrogel via a Schiff base reaction, enabling the slow release of both peptides from the composite hydrogel scaffold. Regarding the osteogenic and angiogenic activities of this hydrogel, in vitro cellular experiments indicated that the composite hydrogel significantly enhanced the osteogenic differentiation of BMSCs and the angiogenic capability of HUVECs, and the in vivo bone regenerative potential of the hydrogel scaffold was determined by establishing a rat critical-sized calvaria bone defect model [44], which showed the synergistic effects of KP and QK in promoting new bone production in the defect areas. Taken together, these studies offer alternative approach to synthesize bioactive peptides-containing hydrogels with desired releasing behavior and controllable degradation to realize synergistic effect in facilitating bone regeneration, which have meaningful reference significance in the design of growth factors (such as VEGF and BMPs) loaded hybrid bone substitutes that are also confronted with the similar drawbacks and dilemmas.

### Bioactive metal ion composite with GelMA hydrogels

With the rapid development of biomaterials used for tissue regeneration, metal ions have increasing application potential in BTE due to the multifunctions involved in osteogenesis, osteoclastogenesis and bacterial inhibition [105]. In bone tissue engineering applications, several metallic trace elements have demonstrated good osteogenic capacity, including zinc ions (Zn^2+^), strontium ions (Sr^2+^), cerium ions (Ce^2+^), and gadolinium ions (Gd^3+^). The osteogenic activity of zinc ions is mainly due to their role as structural components of alkaline phosphatase (ALP); in contrast, the osteogenic capacity of other metal ions (Sr^2+^, Ce^2+^, Gd^3+^) is mainly due to their ionic radius being similar to that of calcium ions and their ability to replace calcium ions in osteoblast-mediated processes [85]. In this subsection, several bioactive metal ions with excellent osteoinductive potential were summarized and discussed as the following.

Zinc ions (Zn^2+^) are an important structural component of ALP, and they play a critical role in determining enzyme activity. Due to the role of ALP in osteogenic activity-promoting phosphate deposition and mineralization, the role of Zn^2+^ in osteogenesis cannot be ignored [113, 114]. As is well known, Zn^2+^ can stimulate the gene expression of the transcription factor RUNX2, promote osteoblast differentiation, and inhibit bone resorption as well as osteoclastogenesis [115]. In addition, Zn^2+^ can optimize the mechanical and biological performances of bioceramics (HA, β-TCP) [116]. As described by Liu et al. an injectable photopolymerizable zeolitic imidazolate framework-8 (ZIF-8)/GelMA composite hydrogel (GelMA-Z) was fabricated after UV exposure [45]. The ZIF-8 nanoparticles loaded in the GelMA hydrogel exhibited good fluidity and photopolymerizability, accompanied by the continuous release of Zn^2+^ and acceptable cytocompatibility. In addition, the expression level of ALP and the extracellular matrix mineralization of rat BMSCs were significantly enhanced in GelMA-Z hydrogels, which also demonstrated effective antimicrobial activity against *Porphyromonas gingivalis,* a species that contributes to the development of periodontitis. Moreover, obviously decreased bacterial infection and adverse inflammation, as well as improved alveolar bone regeneration, were observed in a rat model.

**Fig. 12:**
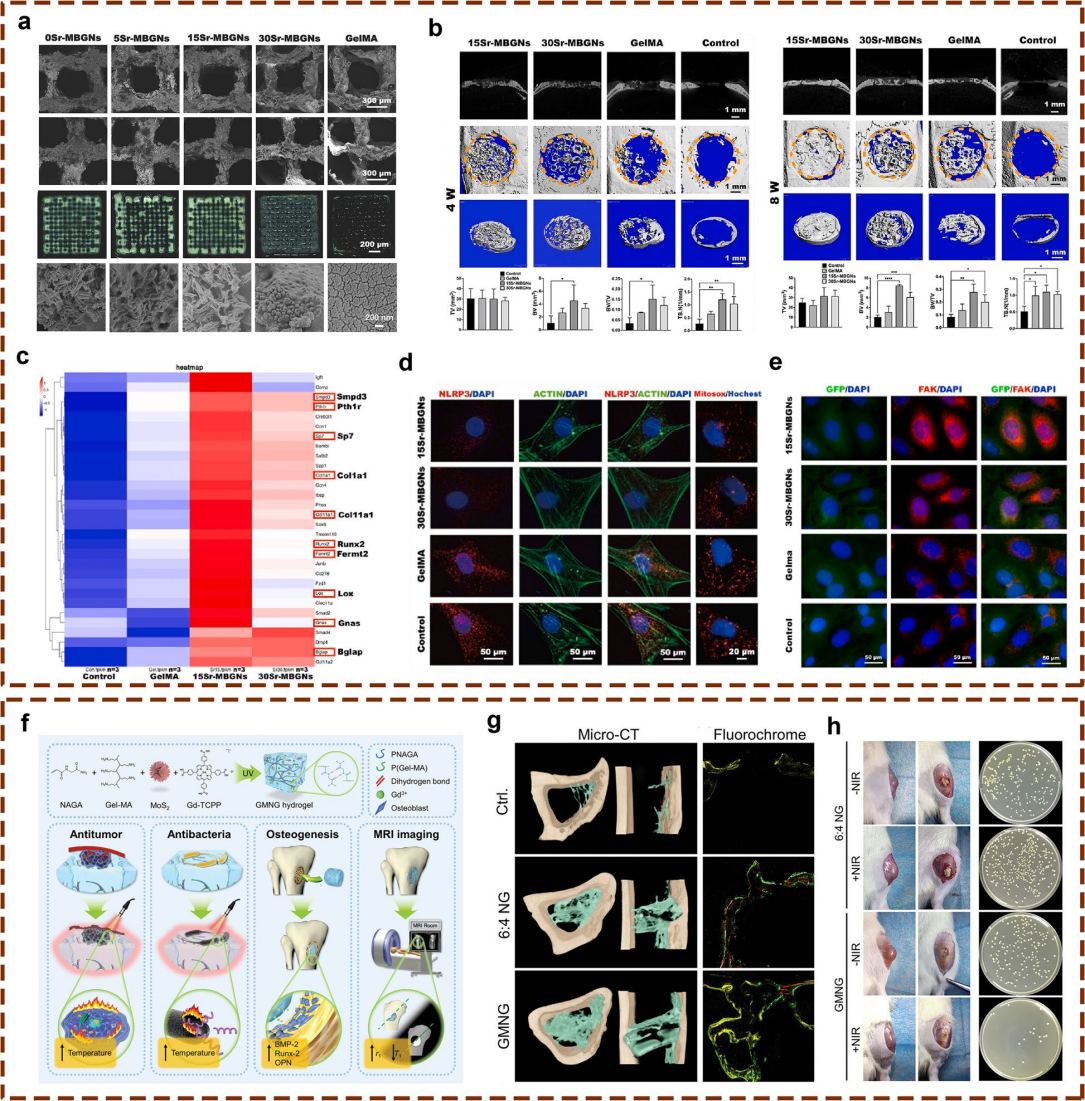
Bioactive metal ion composite with GelMA hydrogels with excellent osteoinductive potential for treating bone defects under specific circumstances. **a** Morphological observations of 3D-printed Sr-MBGN-loaded hydrogel scaffolds. **b** Micro-CT evaluation of in vivo bone healing in a type II diabetic rat critical-sized bone defect model with 3D-printed Sr-MBGN-loaded hydrogel scaffolds at 4 and 8 weeks after implantation. **c** The related upregulated genes collected from the GO enrichment of osteogenesis function presented in the heatmap. **d** The immunoregulatory effect of Sr-MBGN-loaded hydrogel scaffolds on TIID BMSCs. **e** GFP and FAK expression representative of angiogenesis in HUVECs with different hydrogel groups. Images reproduced from [46], © 2023 Elsevier Ltd. **f** Schematic description of the fabrication and multifuntionality of GMNG hybrid hydrogel scaffold. **g** New bone formation induced by GMNG hydrogels as determined by micro-CT reconstruction and fluorochrome-labeling analysis at 12 weeks after implantation. **h** The in vivo photothermal anti-infective capability of GMNG hydrogel in infectious bone defects. Images reproduced from [48], © 2023 Wiley–VCH GmbH

Strontium promotes bone regeneration by activating calcium-sensitive receptors (CASR) in osteoblasts, stimulating osteoprotegerin (OPG) production, and suppressing the expression of nuclear factor κβ ligand receptor activator (RANKL), thereby inhibiting RANKL-induced osteolysis [117, 118]. To exploit the ability of strontium to promote osteogenesis, Xu et al. introduced a bioactive scaffold composed of GelMA and strontium-containing mesoporous bioactive glass nanoparticles (Sr-MBGNs) [46] (Fig. 12a-e). The Sr-MBGNs acted as biomineralization precursors, releasing Sr, Ca and Si ions, inducing the orderly formation of HAP and promoting osteogenesis. In vivo experiments confirmed that the scaffold increased the level of OCN (NCPs), regulated the alignment of hyaluronan in intralaminar mineralization and promoted osteoblast differentiation via the Kindlin-2/PTH1R/OCN axis. This study suggested that this multifunctional biomineralization- inspired platform improved the diabetic microenvironment, signifying its application potential in combating diabetic bone injury with chronic inflammation.

The ionic radius of the cerium (Ce) ion is very close to that of the calcium ion, and the substitution of Ce ions facilitated the biological and mechanical performance of HAP. Previous studies have confirmed the positive role of Ce ions in the proliferation, differentiation, and mineralization of osteoblasts [119, 120]. The accumulation of ROS in bone defects results in impaired cellular viability and restricted new bone formation, and engineered bone scaffolds endowed with osteoinductive and antioxidant properties are urgently needed to improve the outcome of bone tissue repair. In this scenario, Kurian et al. prepared hybrid matrices (Ce@GelMA) composed of GelMA and nanoceria (nCe) through sequential in situ deposition and cryodesiccation [47]. The mechanical properties, such as stress relaxation and compressive modulus, and the physiological stability of GelMA were significantly enhanced after the surface modification of nCe. Furthermore, rapid capture of detrimental ROS and enhanced growth and proliferation of rBMSCs were observed in Ce@GelMA hydrogel scaffolds, confirming the ROS scavenging ability of loaded nCe and its good cytocompatibility with osteogenic cells. Therefore, the Ce@GelMA hydrogel scaffolds are an engineered nanoplatform with the potential to supports acellular mineralization and ROS responsiveness to guide bone regeneration.

Gadolinium (Gd), as a rare earth element, has a similar ionic radius to calcium ions that exhibits good bone-building capacity. It also demonstrated the advantage of improving the physical properties and making the material luminescent for imaging [121]. Moreover, Gd complexes are highly effective magnetic resonance imaging (MRI) contrast agents that are widely used in clinical practice. In one study, the above properties of Gd were utilized by introducing it into a GelMA hydrogel system to prepare a multifunctional bone repair material, a Gd-complex and molybdenum sulfide (MoS_2_)-codoped N-acryloyl glycinamide (NAGA)/GelMA multifunctional hydrogel (GMNG) [48] (Fig. 12f-h). Based on the MRI effects of the Gd complex, the position and degradation situation of the hydrogel can be monitored. Moreover, the incorporation of MoS_2_ nanoparticles endows the GMNG hydrogel with excellent photothermal ability, leading to outstanding antimicrobial and antitumor properties. Additionally, the slow release of Gd^3+^ from the GMNG hydrogel with the gradual degradation of GelMA network facilitated the in vitro osteogenesis and in vivo regeneration of bacterial infected bone defects. Interestingly, the in vivo degradation behavior of implanted GMNG hydrogel during the bone healing process could also be monitored with the aid of the unique magnetic property for MRI scanning. Therefore, this composite GMNG hydrogel combines antitumor, antibacterial, MRI, and osteogenic functions in one package, providing a therapeutic basis for the treatment of bone tissue defects caused by bone tumors with high risk of bacterial infection after radical excision.

### Composite hydrogels incorporating 2D nanomaterials

Apart from the composite GelMA hydrogels integrated with bioactive substitutes as summarized aforementioned, there is also an increasing interest in fabricating various of nanomaterials into GelMA network to generate nano-structured hybrid hydrogels with significantly improved physical properties and biological performances [9]. A number of typical 2D nanomaterials, including GO nanosheets, BP nanosheets and NTs, have sparked substantial interest among scientists focused on tissue engineering owing to their unique 2D structures and outstanding physicochemical properties, such as excellent electrical conduction, good biodegradability and available functionality [49, 122]. Based on these unique features, several novel GO-based nanoplatforms, such as copper and gallium nanoparticle-decorated GO nanosheets (GO/Cu and GO/Ga) have been previously prepared and reported by our research team and have demonstrated great therapeutic potential in skin or bone tissue repair in bacteria-infected environments [122–124]. In recent years, these 2D nanomaterials have demonstrated promise for biomedical applications in drug delivery, bone repair and photothermal treatment. Considering their outstanding osteoinductive properties, GelMA composite hydrogel scaffolds loaded with these 2D nanomaterials were fabricated to improve the biomechanical properties and bone healing capacity of the resulting biomimetic substitutes.

#### GelMA/GO nanosheet hydrogels

GO nanosheets, a representative 2D nanomaterial, possess outstanding mechanical properties, electrical conductivity, a large specific surface area (SSA) and a stable atomic structure. These materials could provide sufficient mechanical properties for bone repair scaffolds, facilitate the required electrical stimulation for cellular osteogenic activity and bone formation, and induce the adsorption of active substances [103]. Owing to its agglomeration-prone nature, GO is less mobile and difficult to directly inject into the body [125] and often requires incorporation with other matrix-based materials, such as GelMA hydrogels. According to an investigation performed by Li et al. the addition of GO-based nanosheets into GelMA further improved the osteogenic performance of hMSCs, and in vitro findings indicated the high viability and metabolic activity of hMSCs encapsulated in the newly developed nanocomposites. The addition of GO significantly accelerated mineralization within the structure containing hMSCs, which was further promoted by replacing GO with silica-coated GO (SiGO). In addition, the results showed that the nanosheets facilitated the synthesis, expression, retention and biological activity of endogenous BMP. The in vivo experiments showed that the new bone volume in the groups treated with GO/GelMA and SiGO/GelMA composites loaded with hMSCs was 108 and 385 times larger than that in the GelMA control group, respectively, highlighting the potential of GO-based nanocomposites for BTE applications [49]. Considering the significant effect of the host immune response on implanted biomaterials, Yu et al. exploited the good biocompatibility and osteoinductive activity of GO by introducing it into a 3D-bioprinted scaffold system encapsulating living cells, and alginate/GO was used as an encapsulation system for rat BMSCs, together with GelMA/HAMA as an encapsulation system for rat BMMs, to form a dual-channel bioprinting system for early immune regulation and osteogenic differentiation [27]. The scaffold composed of GelMA/HAMA/alginate/GO was structurally stable, mechanically strong, and biocompatible. In vitro crosstalk experiments with both types of cells showed that BMSCs facilitated the early polarization of BMMs to the M2 type, decreased the expression of proinflammatory genes and increased the expression of anti-inflammatory genes, while BMMs could also promote the osteogenic differentiation of BMSCs. An in vivo rat cranial defect model also showed that dual-channel scaffolds encapsulating BMMs and BMSCs were more effective than single-cell-type scaffolds and decellularized scaffolds, and this novel bioengineered platform may be an effective approach for regulating the early immune microenvironment and late osteogenesis induction within bone defects.

#### GelMA/BP nanosheet hydrogels

**Fig. 13:**
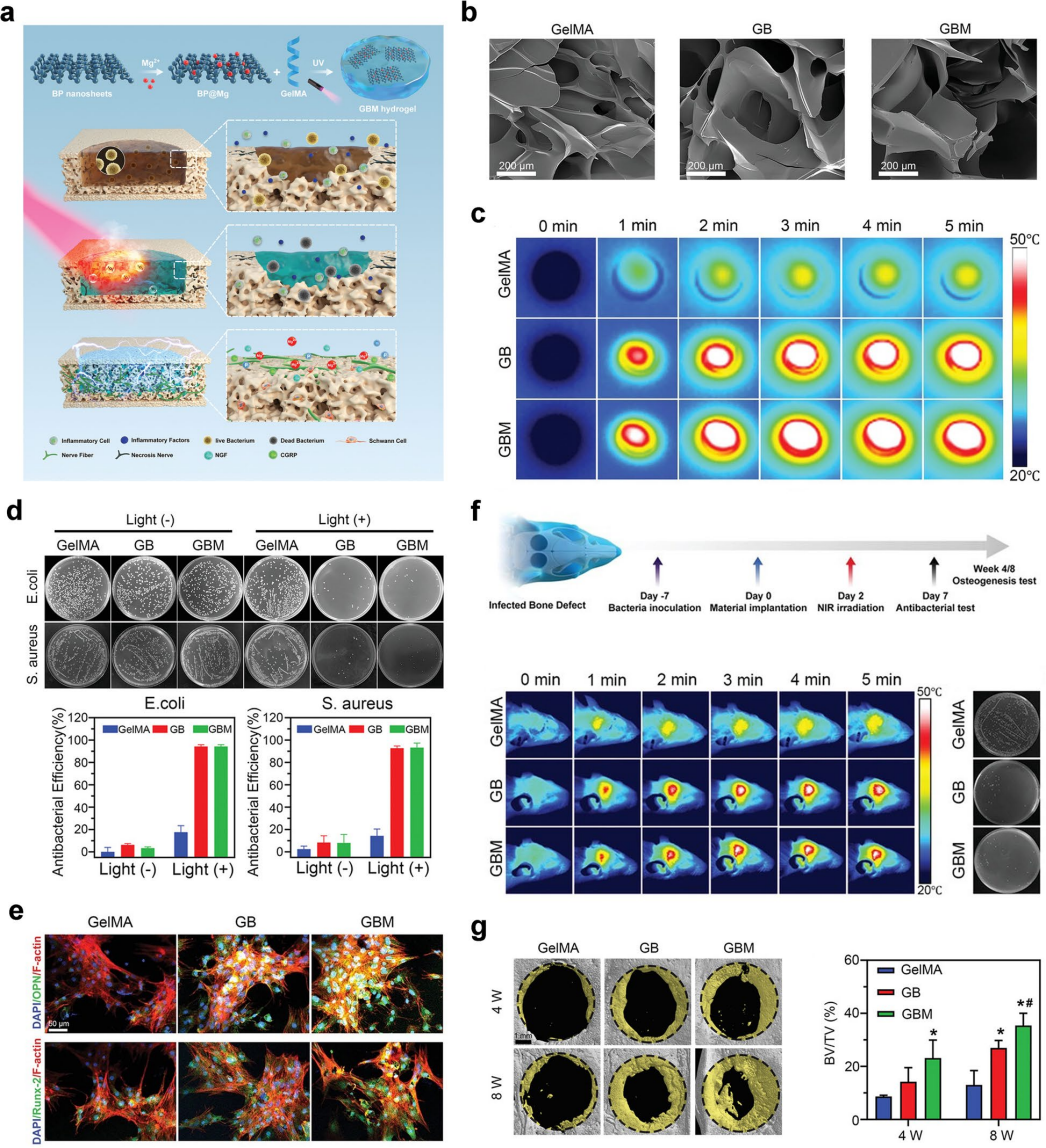
Photosensitive and conductive hydrogel incorporating BP@Mg into GelMA for antimicrobial and innerved bone regeneration of infected bone defects. **a** Schematic illustration of the GelMA-BP@Mg (GBM) hydrogel. **b** SEM images of different hydrogel scaffolds. **c** Photothermal properties of the GelMA-BP@Mg (GBM) hydrogel scaffolds subjected to NIR irradiation. **d** Evaluation of the photothermal antibacterial properties of the GBM hydrogel in vitro*.* **e** In vitro osteogenic potential of the GBM hydrogel via OPN and Runx-2 immunofluorescence staining*.* **f** Schematic description of the experimental process for establishing rat models with infected skull defects. In addition, in vivo infrared thermographic photographs and antibacterial properties of the hydrogels after NIR laser irradiation were performed. **g** Micro-CT images and quantitative analysis of infected bone defects treated with hydrogels at weeks 4 and 8. Images reproduced from [50], © 2022 Wiley–VCH GmbH

As another typical 2D nanomaterial composed of phosphorus, BP nanosheets demonstrate good biodegradability, cytocompatibility, biocompatibility, outstanding electrical conductivity and highly responsive photothermal effects, making them a promising bioactive component for synthesizing multifunctional biomaterials used for different biomedical purposes (such as anti-infection, bone repair and tumor elimination). [126]. In addition, BP has high oxygen reactivity, and the reaction produces phosphate; both BP and phosphate achieved better in situ biomineralization by capturing free calcium ions in the osteogenic microenvironment. Furthermore, BP can directly stimulate the osteogenic differentiation of stem cells through the BMP-2 pathway to accelerate bone regeneration [127, 128]. Due to its good electrical conductivity, BP plays a critical role in promoting neural repair and has been confirmed to promote the differentiation of BMSCs into neuron-like cells [129]. However, BP has poor stability and is prone to oxidation and degradation by reactions with oxygen and water, while the addition of metal ions can increase its stability. Magnesium ions (Mg^2+^) are often applied in bone defect repair, mainly due to their ability to enhance bone activity, which facilitates the osteogenic differentiation of BMSCs and the production of calcitonin gene-related peptide (CGRP) locally in nerves. Moreover, BP nanosheets modified by Mg^2+^ have increased stability and can facilitate neural repair capacity [40]. Jing et al. prepared a BTE material based on magnesium ion-modified BP nanosheets (BP@Mg), clearly demonstrating the ability to promote bone-associated nerve repair and high antimicrobial activity [50] (Fig. 13). The photoresponsive conductive hydrogel was prepared by incorporating BP@Mg into the GelMA hydrogel. The high electrical conductivity of BP@Mg and corresponding bioactive ions released from the hydrogels could synergistically improve the migration and secretion of Schwann cells and promote neurite growth and internal bone regeneration. In vivo experiments showed effective antibacterial activity and enhanced bone and CGRP nerve fiber regeneration in a rat-infected cranial defect model, and these phototherapeutic conductive scaffolds can pave the way toward generating alternative BTE substitutes based on skeletal-related innervation for repairing challenging bacteria-infected bone defects.

#### GelMA/nanotube hydrogels

Nanostructured biomaterials are regarded as promising candidate BTE materials because their nanostructural hierarchy is similar to that of bone tissue [130]. NTs, an additional typical 2D nanomaterial, such as carbon NTs (CNTs), titanium dioxide (TiO_2_) NTs, and halloysite NTs (HNTs), have an extensive range of applications in bone tissue repair engineering because of their unique physicochemical characteristics. CNTs have a tubular structure made of a layer of graphene rolled into a cylinder, and each carbon atom of CNTs is bonded by *sp*^2^ hybridization, which endows complexes with excellent mechanical, electrical, optical, and thermal properties [131]. According to a report by Shin et al. a GelMA hydrogel system containing CNTs was prepared, a thin layer of GelMA was coated on CNTs to improve their biological properties, and then the GelMA-coated CNTs were incorporated into the hydrogel structure without affecting their porosity and cytocompatibility [51]. More importantly, the incorporation of CNTs enhanced the mechanical properties of the hydrogel, which could be tuned by controlling the CNT incorporation amount. In addition, high levels of cell viability, elongation, and proliferation were observed when NIH-3T3 cells and hMSCs were encapsulated within these hybrid hydrogels. The above results suggested that the CNT-GelMA bone substitute with adjustable mechanical properties and satisfactory cell activity can serve as a 3D tissue engineering scaffold material [51]. Some studies have shown that ceramics with different nanostructures (e.g., TiO_2_) exhibit favorable effects on the bone growth rate and formation capacity, and other studies have shown that anodized TiO_2_ NTs induce the enhanced growth and accelerated bone differentiation of MSCs, demonstrating a wide range of applications in bone repair [52, 130, 132]. The aforementioned CNTs and TiO_2_ NTs, both showed deficiencies in composition and degradability, whereas HNTs, as naturally occurring aluminosilicate NTs, showed good biocompatibility and functionality. The nanotube structure of HNTs with good bone regeneration potential has greater potential for application in BTE research [133]. An HNT-encapsulated hydrogel was prepared by the photopolymerization of GelMA and HNTs, and the experimental results showed that the addition of HNTs improved the mechanical properties of the composite while maintaining good in vitro cytocompatibility. The addition of HNTs significantly facilitated the osteogenic differentiation of human dental pulp stem cells (hDPSCs), followed by significant upregulation of the expression of osteogenic differentiation-related genes and accompanying proteins. In vivo rat cranial defect experiments also showed clearly enhanced new bone formation in bone defects implanted with hydrogels containing HNTs [53], presenting a promising bone regenerative strategy for repairing bone defects based on HNT-incorporated GelMA hydrogels.

## 3D-bioprinted GelMA-based bone scaffolds

**Fig. 14:**
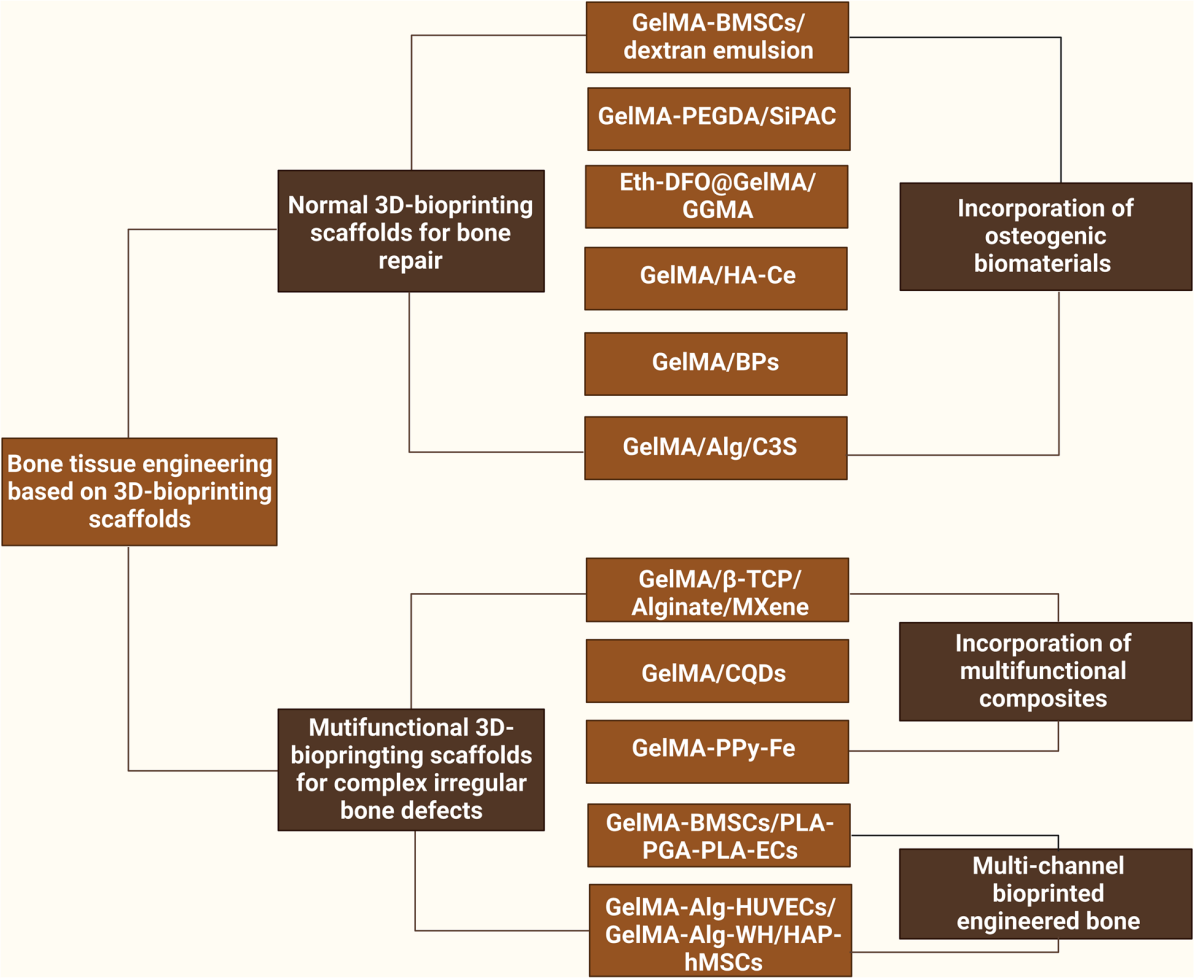
Schematic description of 3D-bioprinted GelMA-based bone scaffolds (created with BioRender.com)

Bioinorganic materials including bioceramics and bioactive glass, are limited in their application to critical size defects in non-load-bearing bones because of their weak mechanical properties [134]. In addition, currently available biomaterials cannot bridge or fill the anatomical shape and structure of lost bone tissue due to the variability of bone defects and thus cannot meet the surgical requirements of larger-sized critical defects. BTE based on 3D-printing technology largely overcomes these difficulties by using available materials to manufacture various novel bone substitutes that provide significantly improved mechanical properties for repairing bone defects with high load-bearing requirements. More importantly, it is practical to fabricate engineered bone substitutes with various geometries using 3D-printing technology, which allows control of the volume, geometry, and internal structure of tissue scaffolds to maximize the match to the bone defect site and improve the bone repair performance of scaffolds [135]. As reported in our previous works, 3D-bioprinting technology has been widely used to prepare porous scaffolds with multifunctional therapeutic properties for treating challenging bacteria-infected bone defects, offering possible advantages over allogenic or xenogenic grafts [136–138]. The rapid gelation, light-curing properties, and biocompatibility of GelMA satisfy the requirements of the currently widely used 3D-printing technologies. As the bioprinting solution is extruded from the machine, it is then subjected to focused UV radiation at the appropriate time points. Subsequently, it is rapidly cured under UV radiation focused at the appropriate location [139]. Therefore, GelMA is often combined with 3D-printing technology for BTE applications to construct corresponding scaffolds for more complex bone defects owing to its excellent plasticity and ability to fit defects (Fig. 14).

**Fig. 15:**
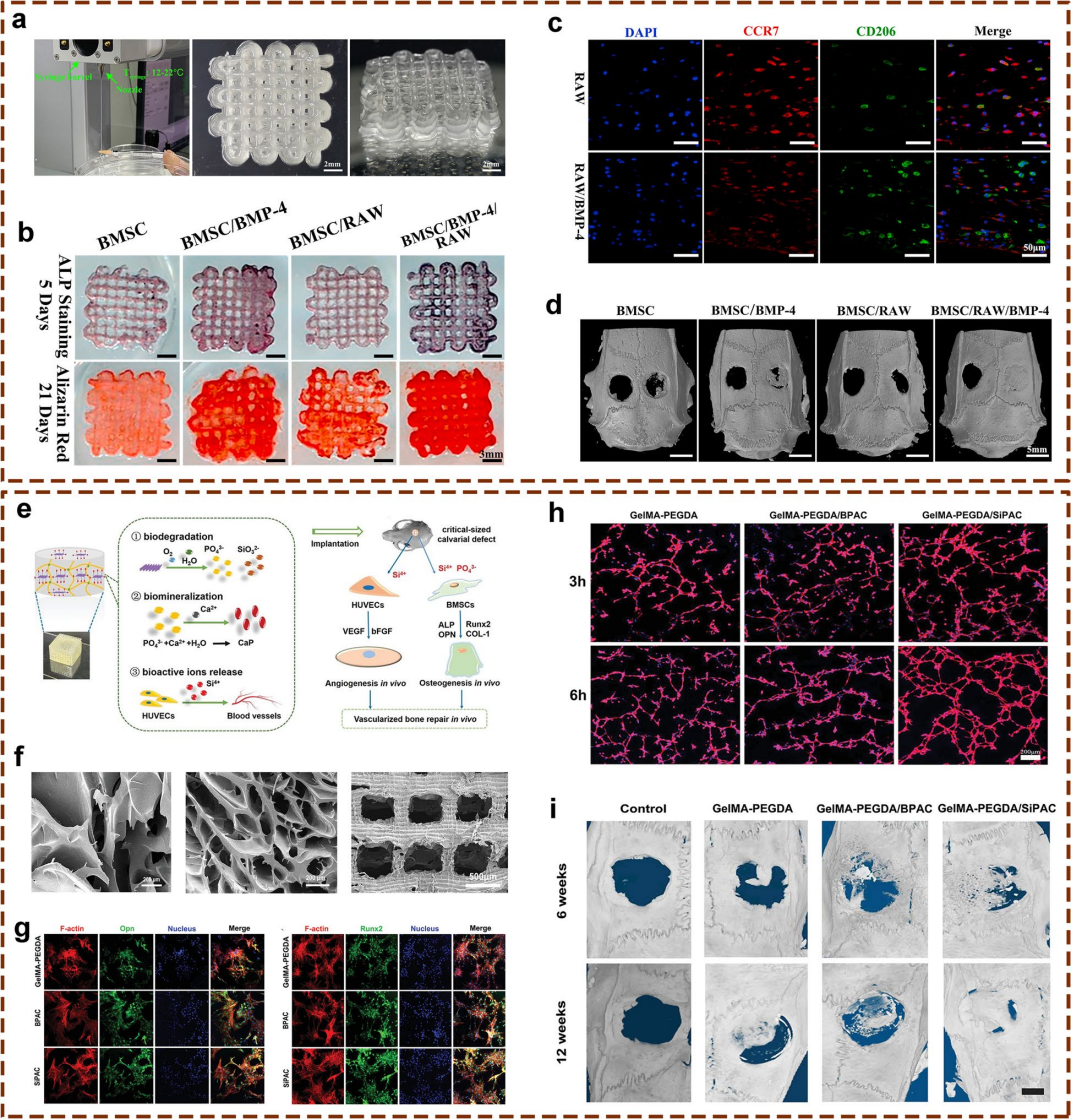
3D-bioprinted hydrogel as bioactive and biodegradable bone scaffolds for accelerated bone regeneration. **a** 3D bioprinting process of GelMA hydrogel integrated with BMSCs, RAW264.7 and BMP-4-loaded MSNs. **b** Osteogenic differentiation of BMSCs in the composite scaffolds. **c** Polarization and inflammatory regulation of loaded RAW264.7 macrophages in the hybrid hydrogels. **d** In vivo therapeutic efficacy of diabetic bone defects induced by the 3D-bioprinted hydrogel scaffolds in a calvarial defect model. Images reproduced from [41], © 2020 Elsevier. **e** Schematic description of released bioactive ions from the 3D-printable GelMA-PEGDA/SiPAC hydrogel scaffolds with improved angiogenesis and osteogenesis for augmented vascularized bone repair. **f** SEM images of the printed hydrogel scaffolds. **g** Significantly increased expression of osteogenic marker genes (OPN and RUNX2) induced by the hybrid hydrogels after immunofluorescence staining. **h** In vitro angiogenesis evaluation of HUVECs cultured on the hydrogel scaffolds. (i) Accelerated bone regeneration of calvarial defects treated with GelMA-PEGDA/SiPAC hydrogel scaffold as shown in micro-CT reconstructed images. Images reproduced from [35], © 2021 Wiley–VCH GmbH

Many of the bone repair materials reported in previous studies were fabricated based on 3D technology. To overcome the disadvantages of the extrusion-based bioprinting method in structural integrity and fabrication speed regarding the preparation of bone substitutes, Tao et al. reported a rBMSC-encapsulated GelMA/dextran emulsion via a digital light processing (DLP)-based 3D bioprinting platform [56]. This 3D hydrogel scaffold facilitated the cell proliferation, migration, spreading and osteogenic differentiation of rBMSCs via regulation of the YAP signaling pathway. In addition, the in vivo bone healing of rat cranial defects confirmed the therapeutic effects of these void-forming hydrogels. As reported by Guo et al. a 3D framework composed of GelMA and nHAP and microspheres made of GelMA and CSA were integrated into the framework as bridges and channels for cell adherence and migration [17]. The addition of GelMA/CSA microspheres significantly promoted the stability and compression properties of the prepared hydrogel scaffolds, and the CSA released from the microspheres evidently facilitated the migration and osteogenic differentiation of osteoblasts, leading to accelerated bone regeneration in a mouse calvarial defect model. These multimodule bioactive hydrogel scaffolds fabricated using 3D printing technology provided a favorable bone tissue microenvironment for the effective interaction of cells and bioactive factors, thereby facilitating the healing of bone defects. Sun et al. used gelatin, GelMA and PEG as matrix bioink to prepare a scaffold loaded with an active factor (BMP-4) as well as living cells (BMSCs) by 3D bioprinting technology. The scaffold exhibited good mechanical properties and further improved cell viability and the inflammatory microenvironment, demonstrating great bone regenerative potential in SD rat with diabetes mellitus (DM) [41] (Fig. 15a-d). Xu et al. prepared 2D SiP nanosheet-enhanced photocrosslinkable and 3D-printable GelMA-PEGDA/SiPAC hydrogels by using ethylene-surface functionalized SiP nanosheets as carriers, and the prepared scaffold had excellent angiogenic and osteogenic abilities [35] (Fig. 15e-i). Li et al. used photocrosslinking and ionic crosslinking techniques combining DFO-loaded Eth with GelMA/GGMA hybrid bioink to fabricate a 3D-printed scaffold, which exhibited good mechanical properties and bone regeneration ability [29]. In addition, Leu Alexa et al. produced 3D printable hydrogels based on GelMA and HA-doped with cerium ions, which displayed high structural integrity and homogeneity and improved osteogenic differentiation [55].

In view of the evident advantage of 3D printing in preparing bone substitutes for repairing infectious bone defects with irregular shapes, Nie et al. reported a GelMA/β-TCP-based hydrogel scaffold modified with customized MXene (Ti_3_C_2_) (GTAM), which showed excellent photothermal antimicrobial and osteogenic capabilities [21]. The cell-laden 3D porous hydrogel scaffolds exhibited excellent photothermal antimicrobial activity and good cytocompatibility, leading to synergistic therapeutic effects on *S. aureus*-infected mandible defects of rats under NIR irradiation. Shen M et al. used 3D bioprinting technology to construct in situ-vascularized tissue-engineered bone, and it was reported that capillary ECs could be cultured under tumor-mediated conditions to form capillary networks, suggesting that ECs can form vascular networks under in vitro conditions [26]. Inspired by this conjecture, the investigators developed a 3D-bioprinted scaffold to uniformly inoculate rat aortic endothelial cell lines (RAOECs) on the surface of a porous scaffold with the aim of forming a scaffold for bone defect repair with effective internal vascularization and osteoinductive activities. This study used two bioprinting inks: GelMA hydrogel, a photosensitive hydrogel that served as a matrix bioink and was loaded with BMSCs for bone regeneration, and PLA/ polyglycolide (PGA)/PLA (3P) hydrogel, a thermosensitive hydrogel, which served as a template bioink and was loaded with ECs for angiogenesis. These two bioinks were bioprinted together line by line in alternating parallel lines to form a monolayer structure and then repeated layer by layer to form a scaffold structure. ECs were adhesively implanted onto the surface of interconnected tubular channels by sacrificing the template bioink after photocrosslinking and then cultured in a perfusion bioreactor to form a vascular network. In vitro experiments showed that the in situ vascularized scaffolds exhibited coupling effects between angiogenesis and osteogenesis. Furthermore, RNA sequencing enrichment analysis showed that these in situ vascularized scaffolds prepared by 3D bioprinting promoted osteogenesis and angiogenesis by increasing the expression of genes in related biological processes [26]. To overcome the therapeutic challenges in the repair of large irregular bone defects, Ghahri et al*.* prepared a biomimetic bone repair scaffold by simulating the bone structural units of dense bone [54]. This 3D scaffold-cell construct is divided into two main parts, the outer osteogenic layer, the inner angiogenic layer and the hollow pipe in the center, similar to the Haversian system, where the outer layer consists of GelMA-alginate hydrogel with hBMSCs and two bone minerals (whitlockite and hydroxyapatite), and the inner layer is composed of GelMA-alginate hydrogel and HUVECs. The bionic scaffold with osteon-like biomimetic microstructure displayed appropriate mechanical strength, stable swelling actions, appropriate degradability properties, satisfactory cytocompatibility and excellent osteogenic properties in vitro. Unfortunately, this study was not validated by further in vivo animal experimental models, but there is much to recommend. Unlike previous bionic scaffolds that mimic the macroscopic structure of natural bone, this study focuses on the microstructure-the bone unit, providing new ideas and methods for subsequent bone tissue engineering research.

**Fig. 16:**
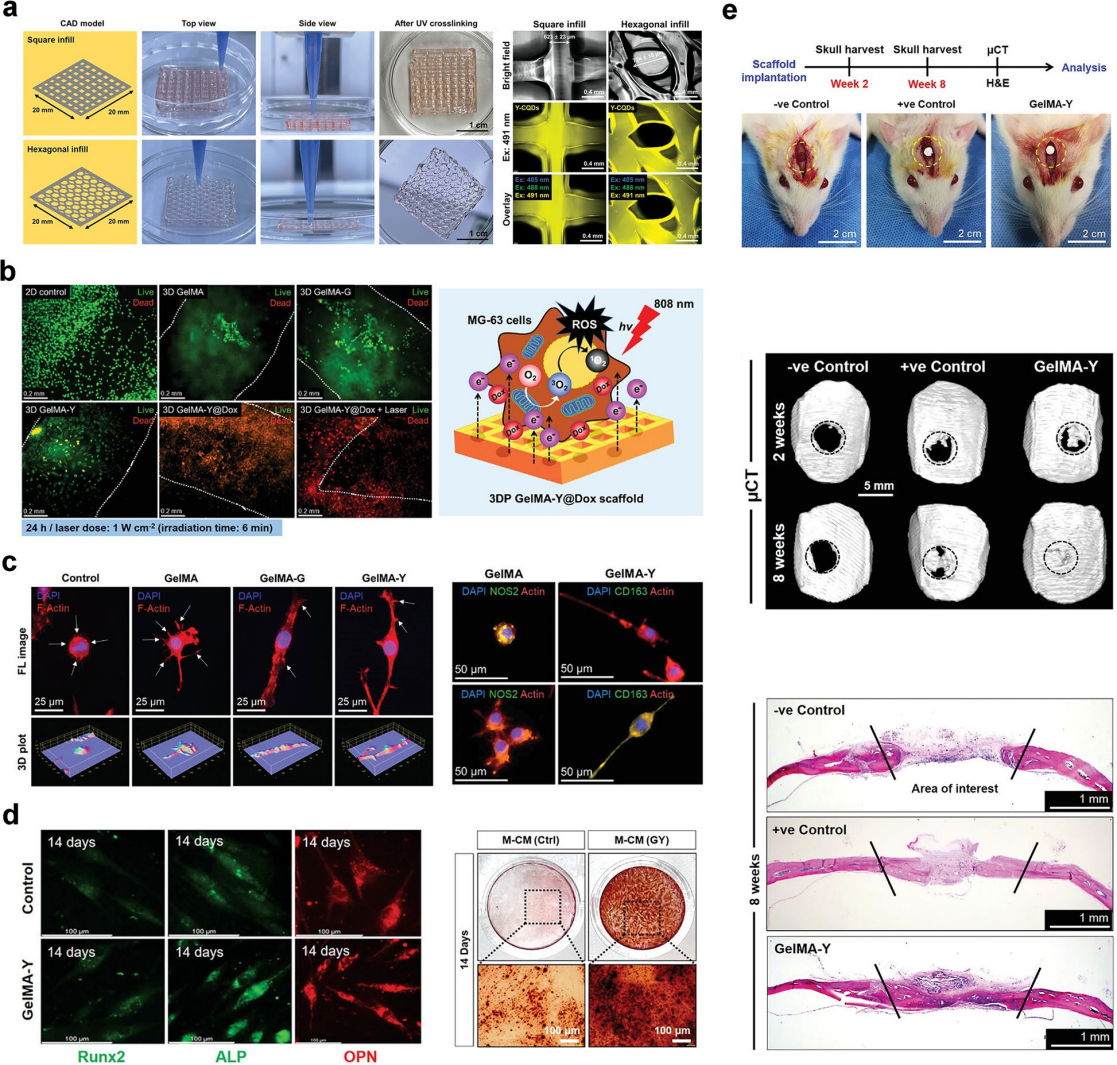
3D-bioprinted nanoengineered hydrogel (GelMA/CQDs) with photoactivated drug delivery for tumor apoptosis and simultaneous bone regeneration via macrophage immunomodulation. **a** The optimized printing parameters for the GelMA-Y bioink. In addition, representative digital photographs and microscopic images of the 3D printed GelMA-Y hydrogels were recorded. **b** Cytotoxicity evaluation of the MG-63 cells after irradiation with an 808 nm NIR laser. A hypothetical drawing of NIR-triggered hypothermia and ROS-induced bone tumor irradiation was also provided. **c** The M2 polarization of Raw 246.7 cells cultured on different hydrogel scaffolds in vitro. **d** In vitro osteogenic properties of hBMSCs after 14 d of incubation in a macrophage-conditioned media(M-CM). **e** In vivo bone regeneration capability of the 3D-printed GelMA-Y scaffolds in a rat skull defect model. Images reproduced from [59], © 2023 Wiley–VCH GmbH

**Fig. 17:**
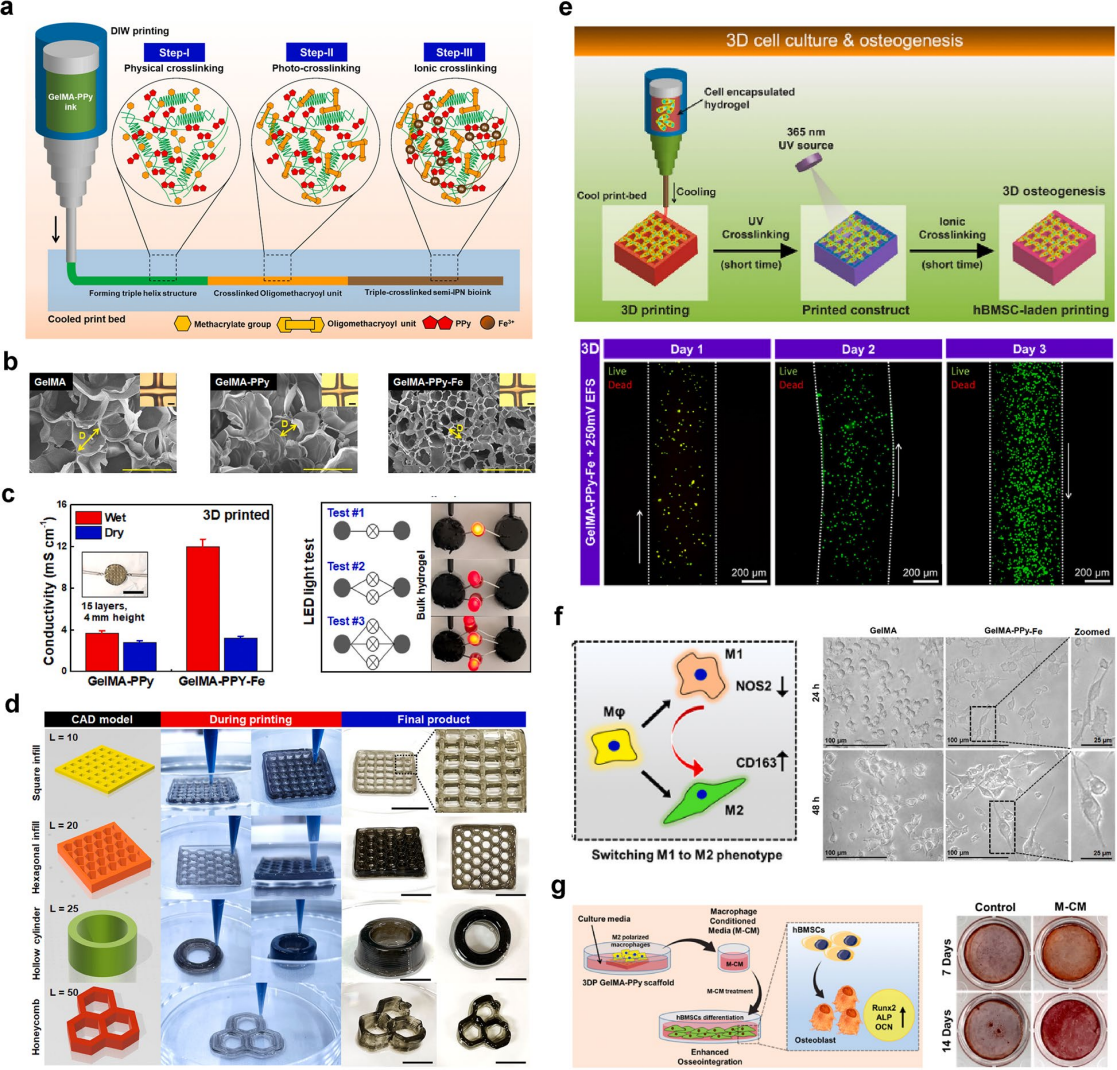
Electrically stimulated 3D-bioprinted GelMA-PPy-Fe hybrid hydrogel induces osteogenesis and bone regeneration by regulating collective signaling pathways and immunopolarization. **a** Schematic illustration of the ink deposition process. **b** FE-SEM images of the freeze-dried GelMA, GelMA-PPy, and GelMA-PPy-Fe hydrogel scaffolds. **c** Conductivity of the GelMA-PPy and GelMA-PPy-Fe hydrogels. **d** Determination of the mechanical integrity of 3D printed complex architectures using GelMA-PPy bioink. **e** Schematic illustration of the cell culture procedure showing the protocol for cell-laden culture (3D), and the cell viability of 3D bioprinted hBMSCs after 5 days of incubation was also determined. **f** Macrophage polarization potential of Raw 264.7 cells cultured with the fabricated scaffolds. **g** Schematic illustration of the osteo- immunomodulation experiment and the ARS staining of hBMSCs in the presence of M-CM after 7 and 14 days of induction. Images reproduced from [60], © 2023 Elsevier Ltd

Considering the importance of the biofunctionality of 3D-bioprinted constructs, Ratheesh et al. developed a bioink based on autologous bone particles (BPs)/GelMA and investigated the rheological properties and printable window of this bioink incorporated with microparticles [57]. The bioink consisted of BPs of various sizes (0–500 μm) in GelMA at different concentrations (from 5 to 15% w/v), and the results showed that 15% w/v BPs loaded at concentrations of 10% and 12.5% GelMA had higher print quality, with both the strong shear thinning behavior and the high gel strength necessary for printing. In addition, in vitro cellular experiments showed that the cells initially contained in the BPs were able to migrate and colonize the bioprint while maintaining their ability to express early osteogenic markers, indicating the potential application of BPs as a personalized therapeutic strategy for more effective bone healing. The reproducible fabrication and maintenance of cell activities within scaffolds remain a great challenge for 3D bone substitutes. According to the work conducted by Beheshtizadeh et al. extrusion-based 3D bioprinting technology was applied to prepare alginate (Alg)/tri-calcium silicate (C_3_S) bone scaffolds modified by GelMA hydrogels, which showed good printability, improved mechanical properties, and satisfactory cytocompatibility and osteogenic capability [58]. This study suggested that GelMA-decorated 3D-bioprinted Alg/C3S scaffolds could be used for large and complicated bone defects via modification of extrusion-based 3D-printed constructs. The repair of bone defects caused by tumor resection is still a significant challenge, and multifunctional hydrogels with osteogenic, angiogenic and antitumor properties are urgently needed. Dutta and the colleagues prepared a 3D printable multifunctional hydrogel scaffold composed of polyphenolic carbon quantum dots (CQDs) and GelMA, which demonstrated satisfactory printability, M2 polarization of macrophages, anti-inflammatory activity, enhanced osteogenesis and angiogenesis, NIR-triggered anti- osteosarcoma performance and vascularized bone regeneration in a rat calvarial defect model [59] (Fig. 16). This study emphasized the significant role of osteogenic and immunomodulatory properties in promoting osteoimmunity-dependent bone regeneration, and the as-prepared GelMA-CQD nanohybrid hydrogel substrate showed great application potential for combating osteosarcoma resection-induced bone defects that require simultaneous tumor elimination and osteogenesis. In another work reported by Dutta et al. a highly feasible 3D-printed hydrogel based on polypyrrole (PPy)-modified GelMA and Fe^3+^ with a thermo-photo-ionic crosslinking strategy was fabricated to resolve the shortcomings of cell-laden constructs in crosslinking strategy, biomechanical strength and print precision [60] (Fig. 17). This stable GelMA-PPy-Fe hydrogel scaffold exhibited excellent shape fidelity, good cytocompatibility and improved osteogenic differentiation of hBMSCs via regulation of the NOTCH/MAPK/SMAD and Wnt/β-Catenin signaling pathways as confirmed by transcriptomic analysis. Therefore, it provided an ideal conductive hydrogel network with excellent mechanical properties, good structural stability and biocompatibility via one-step in situ biofabrication for effective bone regeneration. Overall, with the rapid progress in manufacturing high-resolution biomimetic biomaterials with excellent printability and bioactive effects, 3D bioprinting technology offers particular insights into the fabrication of bone scaffolds with precise, personalized and diverse models to target various bone defects.

## Conclusion and prospects

For all types of bone defects commonly seen in clinical practice, traditional treatment modalities, including autologous or allogeneic bone graft transplantation, are often limited due to various potential risks, such as donor site injury, limited availability, potential infectious disease transmission, immunological rejection and weighty economic burden [140]. Therefore, it is particularly urgent and challenging to develop synthetic bone grafts for effective bone defect treatment. The application of such BTE materials to mimic the structural, mechanical and biological properties of natural bone to better repair bone defects and provide better treatment for patients with bone defects is the current purpose of most BTE research. With the continuous efforts to develop BTE-based technology, studies on GelMA-based hybrid hydrogel substitutes account for a considerable proportion of the various BTE studies [9, 141]. In this study, we reviewed various types of bone repair materials with different biological functions based on the GelMA hydrogel system. First, inorganic materials with osteoconductive properties, such as bioceramics and BG materials, and cell-carrying scaffolds with strong biocompatibility were used to improve the osteoconductivity of the bone scaffolds. Second, biomaterials with angiogenic properties, such as bioinorganic ions and nanocomposites doped with vasoactive factors or simulated biomimetic bone membranes, are commonly applied to achieve vascularized osteogenesis and bone regeneration. Third, biomaterials with osteoinductive or osteopromotive capabilities were incorporated into the GelMA-based platforms to enhance the osteoinductivity of the implanted materials. In addition, 3D-bioprinted bone repair scaffolds have been shown to be a good choice for treating irregular bone defects and load-bearing bone damage.Notably, several prepared hydrogel scaffolds summarized in this review have a certain similarity, as mentioned above; therefore, multifunctional bone substitutes were fabricated via direct structuring, template synthesis or 3D bioprinting technology to meet various requirements for bone defect repair under different circumstances. Considering the rapid gelation and photocrosslinking properties of GelMA hydrogels, their controllable material structure and enhanced mechanical properties are useful for direction for treating complex bone defects.

GelMA hydrogels stand out from other types of hydrogels because of their photocrosslinking properties. Nevertheless, the biosafety of GelMA-based hydrogels, especially viable cell-laden scaffolds, remains a major obstacle to clinical application, mainly due to their photocrosslinking under UV radiation, which may damage cellular DNA through oxidative stress and may even cause mutations. Thus, it is necessary to improve GelMA gelation methods, and more effective and user-friendly technologies need to be developed and validated. Despite the significant application prospect of GelMA-based hybrid hydrogels as attractive substitutes candidate for BTE, the long-term in vivo degradation, cytotoxicity or biosafety of nanocomposites or metal ions incorporated into GelMA hydrogels should also be scrupulously and objectively investigated in future research, and alternative strategies are highly recommended to achieve required biological effects with good biocompatibility at low concentration of incorporated nanomaterials or metal ions. Notably, cell differentiation within GelMA hydrogel systems that encapsulate cells requires particular attention and in-depth verification, and the in vivo stability of 3D bioprinted cell-hydrogel composites remains to be validated in various types of animal models. In addition, the elasticity modulus and flexibility of biodegradable GelMA-based hybrid hydrogel scaffolds reported up till now are still relatively inadequate to well match with the natural biomechanical properties of human tissues, leading to unavoidable stress shielding, thus, as a bone defect repair material, GelMA hydrogels still need to be further improved in terms of their softness, strength and mechanical support properties. In consideration of the rising concerns about the long-term instability and possible inactivation of loaded bone growth factors within the composite GelMA-based hydrogels as discussed previously, more detailed in vitro and in vivo experiments regarding the encapsulation technology and delivery system of growth factors, as well as actual metabolism in living microenvironment are also required. The majority of the current research is performed using relatively small-sized animal models involving critical bone defects, such as cranial defects and femoral condyle defects in mice, rats or rabbits, while for large bone defect repair in large-scale models, the mechanical properties of the GelMA hydrogel scaffold alone may be insufficient, so the means to incorporate other materials that can significantly enhance mechanical properties should be given more attention. The discovery of piezoelectricity, endogenous electric fields and transmembrane potentials in several biological tissues, such as bone, tendon, ligaments, cartilage, skin and collagen, fostered the concerns about the significant role of electric fields in cell function, which further accelerated the development of bioengineered technologies via electrical stimulation for tissue regeneration [142, 143]. Therefore, GelMA hydrogels integrated with conductive substances open promising approach to synthesize multifunctional tissue constructs with simultaneous biocompatibility and piezoelectric properties, and hybrid hydrogels with piezoelectricity and minimal toxicity may be the next generation of BTE scaffolds for alternative therapy of bone tissue injury. In summary, with the rapid development of BTE, effective countermeasures as discussed above should be proposed to improve mechanical properties, degradation behaviors, microstructure, bioactive functions and long-term biosafety. Regardless of those challenges, numerous advancements of GelMA-based BTE technologies will be continually introduced, and the GelMA-based hydrogel substitutes seem to hold promise for the clinical treatment of bone defects in near future.

## Data Availability

All data is available upon request to the corresponding authors.

## Abbreviations

ABMSCs: Alveolar bone mesenchymal stem cells
AC: Acryloyl chloride
ADSCs: Adipose mesenchymal stem cells
Alg: Alginate
ALP: Alkaline phosphatase
BG: Bioglass
BMMs: Bone marrow-derived macrophages
BMPs: Bone morphogenetic proteins
BMSCs: Bone marrow mesenchymal stem cells
BP^a^: Bisphosphonate
BP^b^: Black phosphorus
BPs: Bone particles
BRUs: Bone regeneration units
BTE: Bone tissue engineering
CaPs: Calcium phosphate nanoparticles
CASR: Calcium-sensitive receptors
CAT: Catalase
CGRP: Calcitonin gene-related peptide
CNTs: Carbon nanotubes
Col I: Collagen I
CPO: Calcium peroxide
CQDs: Carbon quantum dots
C_3_S: Tri-calcium silicate
CSA: Chondroitin sulfate A
DBM: Decalcified bone matrix
DFO: Deferoxamine
DLP: Digital light processing
DM: Diabetes mellitus
ECs: Endothelial cells
ECM: Extracellular matrix
e-NOS: Endothelial nitric oxide synthase
EPCs: Endothelial progenitor cells
Eth: Ethosomes
GelMA: Methacrylate-based gelatin
GGMA: Gellan gum methacrylate
GO: Graphene oxide
HA: Hyaluronic acid
HAD: Dopamine-modified HA
HADSCs: Human adipose-derived stem cells
HAMA: Methacrylated hyaluronic acid
HANFs: HA-nanofibrous scaffolds
HAP: Hydroxyapatite
hBMSCs: Human BMSCs
HCA: Hydroxycarbonate apatite
HDPSCs: Human dental pulp stem cells
HG: High-glucose
HIF-1α: Hypoxia-inducible factor 1-α
hMSCs: Human mesenchymal stem cells
HNTs: Halloysite nanotubes
HUVECs: Human umbilical vein endothelial cells
mBMSCs: Mouse bone mesenchymal stem cells
MBNs: Mesoporous BG nanoparticles
m-HANFs: Mineralized HANFs
micro-CT: Micro-computed tomography
MMP: Matrix metalloproteinase
MnSOD: Manganese superoxide dismutase
MRI: Magnetic resonance imaging
MSCs: Mesenchymal stem cells
MSNs: Mesoporous silica nanoparticles
NCe: Nanoceria
nHAP: Nanohydroxyapatite
NIR: Near infrared ray
NSCs: Neuroectodermal stem cells
NT: Nanotubes
OCN: Osteocalcin
OGP: Osteogenic growth peptide
OMPs: Oxygen-generating microparticles
OPG: Osteoprotegerin
OSX: Osterix
PCL: Polycaprolactone
PEG: 4-Arm-poly(ethylene glycol) acrylate
PEGDA: Polyethylene glycol diacrylate
PGA: Polyglycolide
pODM: Preosteoblast-derived matrix
PLA: Poly lactic acid
PNS: Peripheral nervous system
PPy: Polypyrrole
PTH: Parathyroid hormone
PTHrp: Human recombinant parathyroid hormone protein
RANKL: Nuclear factor κβ ligand receptor activator
RAOECs: Rat aortic endothelial cell lines
rBMSCs: Rat bone marrow mesenchymal stem cells
RF: Riboflavin
RGD: Arg-Gly-Asp peptides
RNA-Seq: Ribonucleic acid sequencing
ROBs: Rat osteoblasts
ROS: Reactive oxygen species
RUNX2: Runt-related transcription factor 2
SBFs: Simulated body fluids
SEM: Scanning electron microscopy
SiGO: Silica-coated GO
SiP: Silica-phosphorus
Sn: Nanosilicate
SNP: Silicate nanoparticles
SSA: Specific surface area
β-TCP: β-Tricalcium phosphate
TGF: Transforming growth factor
TRAP: Tartrate-resistant acid phosphatase
USCEXOs: Human urine-derived stem cell exosomes
UV: Ultraviolet
vECM: Vascular-derived ECM
VEGF: Vascular endothelial growth factor
